# Testing and treating anaemia in pregnant women in Bangladesh: a cross-sectional survey

**DOI:** 10.1136/bmjph-2024-002167

**Published:** 2025-07-16

**Authors:** Ebony Verbunt, Mohammed Imrul Hasan, Eliza M Davidson, Shamim Ahmed, Alistair R D McLean, A M Quaiyum Rahman, Mohammad Saiful Alam Bhuiyan, S M Mulk Uddin Tipu, Bidhan Krishna Sarker, Jena D Hamadani, Sant-Rayn Pasricha, Cathy Vaughan, Meghan A Bohren, Khic-Houy Prang

**Affiliations:** 1 Centre for Health Policy, The University of Melbourne School of Population and Global Health, Melbourne, Victoria, Australia; 2 International Centre for Diarrhoeal Disease Research Bangladesh, Dhaka, Bangladesh; 3 Population Health and Immunity division, Walter and Eliza Hall Institute of Medical Research, Melbourne, Victoria, Australia; 4 Methods and Implementation Support for Clinical and Health (MISCH) research Hub, The University of Melbourne Faculty of Medicine Dentistry and Health Sciences, Melbourne, Victoria, Australia; 5 Diagnostic Haematology, The Royal Melbourne Hospital, Melbourne, Victoria, Australia; 6 Gender and Women’s Health Unit, Nossal Institute for Global Health, The University of Melbourne School of Population and Global Health, Melbourne, Victoria, Australia

**Keywords:** Cross-Sectional Studies, Public Health, Female

## Abstract

**Introduction:**

Anaemia during pregnancy is a significant public health problem, disproportionately affecting women in low-and middle-income countries. In Bangladesh, anaemia affects 38.6% of pregnant women. Although much is known about the prevention of anaemia in pregnancy, it is unknown how women are tested and treated. This study therefore aimed to describe how pregnant women in Bangladesh are tested for anaemia, including variations by social and equity dimensions. We also aimed to explore how women are treated for anaemia and use of intravenous iron in pregnancy.

**Methods:**

An interviewer-administered cross-sectional survey was conducted in Narayanganj district, Bangladesh, with analysis limited to women who had previously been pregnant (n=1000). Outcomes of interest were anaemia testing, anaemia treatment, and intravenous iron use. We undertook descriptive analyses and derived estimates and 95% confidence intervals of the percentage of pregnant women tested for anaemia stratified by education, wealth, and person making decisions around women’s healthcare.

**Results:**

Approximately half of women were tested for anaemia in their previous pregnancy (505/963, 52.4%), with lower testing in women with no education (15.8%, 95% CI: 6.0 to 31.3), whose husband had no education (25.6%, 95% CI: 18.2 to 34.2), or in the poorest wealth quintile (34.0%, 95% CI: 27.4 to 41.2). Pregnant women were most commonly tested for anaemia at a private health facility (393/505, 77.8%), by a medical technologist (350/505, 69.3%), using venous full blood count (484/505, 95.8%). Most women diagnosed with anaemia in their previous pregnancy received treatment (135/142, 95.1%). In any prior pregnancies, a small number of women received intravenous iron (33/985, 3.4%).

**Conclusion:**

Our study reveals the need for substantial investments to ensure all women in Bangladesh are tested for anaemia in pregnancy. This could be achieved through increasing point-of-care testing in antenatal care. However, health facilities and health workers may need to be prepared for an increase in women diagnosed with anaemia.

**WHAT IS ALREADY KNOWN ON THIS TOPIC**

- Anaemia during pregnancy is a significant public health problem, affecting 35.5% of pregnant women globally.
- In Bangladesh, 38.6% of women experience anaemia in pregnancy, despite iron and folic acid supplements provided for prevention and treatment for free as part of routine antenatal care.
- Limited evidence exists on how women in Bangladesh are tested and treated for anaemia in pregnancy and use of intravenous iron.

**WHAT THIS STUDY ADDS**

- This study reveals that only about half of women were tested for anaemia in their previous pregnancy.
- This study demonstrates inequities in anaemia testing rates among pregnant women in Bangladesh. It suggests that women who were more likely to be anaemic (e.g., those with no education or poorest) were less likely to be tested.
- Most pregnant women were tested for anaemia using venous full blood count.
- We show that most pregnant women received treatment after being diagnosed with anaemia.
- We found that some women had received intravenous iron in prior pregnancies, including in the primary health care system.

**HOW THIS STUDY MIGHT AFFECT RESEARCH, PRACTICE OR POLICY**

- Substantial investments are needed to ensure all women in Bangladesh are tested for anaemia in pregnancy and could include increasing point-of-care testing in antenatal care.
- Qualitative research with women and health workers is required to understand why women, particularly with no education, whose husband has no education, or in the poorest wealth quintile are not tested for anaemia in pregnancy.
- Findings from our study may inform the design and implementation of strategies and programmes aimed at reducing the significant burden of anaemia among pregnant women in Bangladesh and other low- and middle-income countries.

## Introduction

 Anaemia in pregnancy is a significant public health problem, affecting an estimated 35.5% of pregnant women globally.^1^ The burden is disproportionately concentrated among pregnant women in low- and middle-income countries, particularly within South-East Asia and Africa.^2 3^ For example, approximately 38.6% of women in Bangladesh experience anaemia in pregnancy compared with 10.2% of women in the United States of America.^1^

Anaemia in pregnant women is defined as a haemoglobin level of less than 110 g/L in the first and third trimester, and below 105 g/L in the second trimester.^4^ Iron deficiency is the primary cause of women experiencing anaemia in pregnancy.^5^ During pregnancy, the expansion of the woman’s blood volume and fetal growth means the physiological demands for iron are high, with iron needed to meet the demands of synthesis for haemoglobin.^6^,^8^ For women in low- and middle-income countries, this is compounded by a greater likelihood of early onset of childbearing, short pregnancy intervals, low coverage of antenatal care, food insecurity, and infections such as malaria and intestinal helminths.^9 10^

A woman experiencing anaemia during pregnancy has profound health, development, and economic consequences that impact the whole of society.^11^ For example, maternal mortality is twice as high in women with severe anaemia compared with women without severe anaemia.^12^ Consequences for the baby include preterm birth, low birth weight, and stillbirth.^13 14^ Babies born to women with anaemia who survive birth may have reduced cognition, poorer growth and development, and be iron-deficient or anaemic themselves.^15^,^17^ Controlling anaemia in women of reproductive age is a global public health and research priority.^12^ In 2012, the World Health Organization (WHO) called on countries to halve anaemia prevalence in women of reproductive age by 2025.^18^ However, no country is on track to achieve this goal, with WHO launching a comprehensive framework to improve prevention, diagnosis, and management of anaemia in 2023.^11^

Pregnant women are recommended to take daily oral iron and folic acid supplements as early as possible following conception.^19^ Broader strategies to prevent anaemia in low- and middle-income countries include fortification of staple foods and improving food security and dietary diversity.^20^ In high-income countries, women are typically tested for anaemia by venous full blood count as part of routine antenatal care.^19^ In settings where laboratory assessment is not available or feasible, a point-of-care capillary haemoglobinometer (e.g., HemoCue) is recommended over a capillary colour scale.^19^

Pregnant women are also recommended to take oral iron and folic acid supplements if diagnosed with anaemia.^19^ Although inexpensive, these supplements require several months of treatment to replenish iron stores.^21^ Various factors may limit their use during pregnancy, including gastrointestinal side-effects, misconceptions and beliefs, and inadequate counselling.^22^,^24^

In high-income countries, intravenous iron is recommended for anaemia treatment when iron and folic acid supplementation is not tolerated by women, in emergency cases, and when women initiate antenatal care later in pregnancy.^25^ Intravenous iron is administered directly into the bloodstream, resulting in limited gastrointestinal side-effects.^26 27^ Modern intravenous iron products, such as ferric carboxymaltose, differ from older intravenous iron formulations in that they can be given in a single administration, with an infusion time of 15 minutes.^28^ Modern intravenous iron products are safe, with severe adverse events rare.^29 30^ Although women in low- and middle-income countries are disproportionately affected by anaemia in pregnancy, intravenous iron intervention as a treatment option is typically not available.

### Context of anaemia management in pregnant women in Bangladesh

**Figure 1:**
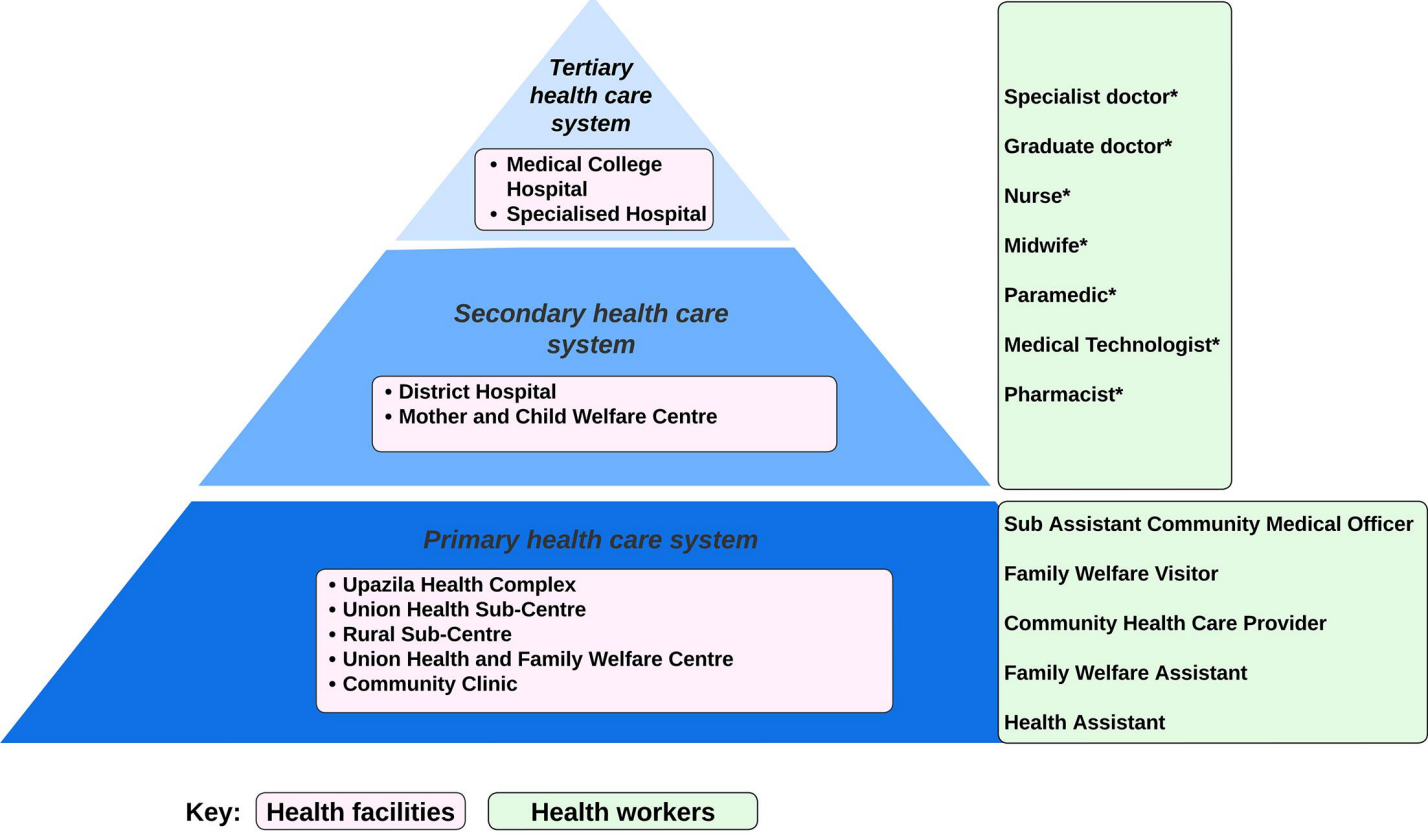
The Bangladesh government health system where pregnant women may receive management for anaemia. *Available at Upazila Health Complex.

In Bangladesh, anaemia in pregnant women is a major public health concern. This is despite iron and folic acid supplements provided for free as part of routine antenatal care in government health facilities.^31^ As shown in figure 1, women may receive antenatal care from government health facilities at the primary, secondary, and tertiary levels of the health care system, with different cadres of health workers at each level.^32^ However, only 33.0% of women receive antenatal care at government health facilities, while 64.0% seek care at private health facilities, 33.0% at home, and 9.0% at non-governmental organisation facilities (the total exceeds 100% as many women receive antenatal care from more than one type of facility during the same pregnancy).^33^ Approximately 20.0% of women adhere to the recommended intake of 180 iron (60 mg) and folic acid (0.4 mg) supplements, with particularly low adherence among women living in social conditions of poverty, with low education, and who experience pregnancy at a young age.^34^ Despite much being known about the prevention of anaemia for pregnant women in Bangladesh, limited evidence exists on how pregnant women are tested and treated for anaemia and use of intravenous iron—which are critical to understanding existing barriers and opportunities for improvement.

### The EDIVA programme

In the context of a persistent high prevalence of anaemia in pregnant women, research institutes in Bangladesh (icddr,b) and Australia (Walter and Eliza Hall Institute of Medical Research (WEHI), University of Melbourne), with the support of the Bangladesh Ministry of Health and Family Welfare, are exploring the potential to implement an intravenous iron intervention in the primary health care system. The EDIVA programme: ‘Efficacy and Demonstration of IntraVenous iron for Anaemia in pregnancy’ aims to assess the effectiveness and feasibility of intravenous iron to treat pregnant women in the second or third trimester with moderate and severe anaemia in Bangladesh. The EDIVA programme has three phases. First, a maternal anaemia prevalence survey.^35^ Second, a randomised controlled trial to determine the efficacy and safety of intravenous iron compared with iron and folic acid supplements (standard-of-care), which is registered on the Australian New Zealand Clinical Trials Registry (ACTRN12621000968875).^36^ Third, a demonstration project to assess the feasibility and acceptability of implementing an intravenous iron intervention in the Bangladesh primary health care system.

Understanding how pregnant women are tested and treated for anaemia in Bangladesh is essential for designing and implementing strategies and programmes, such as EDIVA, that may reduce the significant burden of anaemia. By analysing data from the maternal anaemia prevalence survey in phase 1,^35^ this study aimed to describe how pregnant women in Bangladesh are tested for anaemia, including variations by social and equity dimensions. We also aimed to explore how women are treated for anaemia and use of intravenous iron in pregnancy.

## Methods

This paper is reported according to the STrengthening the Reporting of OBservational studies in Epidemiology statement (STROBE; online supplemental appendix A).^37^

### Study design and setting

This is a cross-sectional survey developed by icddr,b, with support from WEHI and the University of Melbourne (online supplemental appendix B).^35^ The primary aim of the survey was to give insight into the prevalence of anaemia and iron deficiency among pregnant women in Bangladesh, and the results have recently been published.^38^ The secondary aim of the survey was to gather information on women’s sociodemographic characteristics, nutritional knowledge, antenatal and childbirth care-seeking practices and challenges, knowledge and existing practices for anaemia management, and childbirth plans. The survey was not formally piloted prior to study commencement; however, it was reviewed extensively by the research team. The survey was conducted in Bangladesh in three upazilas (sub-districts) of the Narayanganj district: Rupganj, Sonargaon, and Bandar. The study area has a population size of approximately 1 400 000 people.^39^ This area is in close proximity to the capital city Dhaka (approximately 30–35 kilometres) and is a semi-urban, industrialised district.^40^ This setting was purposively selected for the EDIVA programme according to strong existing relationships with the community and local health authorities.^41 42^ Recruitment and data collection took place between October 2021 and April 2022.

### Participants and sample size

Women were included if they were 13–32 weeks pregnant (based on last menstrual period) at the time of survey administration and were residents of Rupganj, Sonargaon, or Bandar upazila. Pregnant women were excluded from participating if they did not provide consent. Based on the primary aim of the survey, a total of 1500 pregnant women were recruited, with a minimum sample size calculation of 600 women per trimester across all upazilas. This sample size was determined to ensure sufficient precision around estimates of the prevalence of anaemia (35.0%, 95% CI: 31.2 to 38.8) and iron deficiency (40%, 95% CI: 36.1 to 43.9) by trimester.^35^

### Data collection

Data were collected regarding women’s current pregnancy (survey sections A–C and E–O) and if applicable, any previous pregnancies (survey section D: pregnancy history). Eligible women were identified from the government Family Welfare Assistant register, where pregnant women in Narayanganj district are identified and recorded. Eligible women were visited at their home by icddr,b field research assistants, and if they met the inclusion criteria, commenced the survey by providing information on their sociodemographic characteristics and nutritional knowledge. The iron levels of the women’s main drinking water source were measured using the HACH iron test kit, Model IR-18B.

Women completed the remainder of the interviewer-administered survey at a nearby Union Health and Family Welfare Centre within 24 hours. Women had the option to be accompanied by a family member, such as their mother-in-law. Women’s anaemia status was tested by measuring haemoglobin levels in venous blood using a HemoCue Hb 301. Anaemia status was explained to the women, and they were provided with iron and folic acid supplements along with usage instructions, as per standard care practices. A Water, Sanitation, and Hygiene (WASH) survey and Edinburgh Postnatal Depression Scale (EPDS) survey were also completed. Data were collected using smartphones with internet-enabled SIM cards. Data were uploaded to an icddr,b database at the end of each day and electronically backed up to servers at WEHI weekly.

### Statistical analysis

To achieve our study aims, we limited our statistical analysis to sociodemographics, pregnancy history, and women’s autonomy (survey sections C, D, and G). As outlined above, women were tested and treated for anaemia in their current pregnancy as part of the survey. Therefore, our analysis focused on women who had been previously pregnant (n=1000) and had been tested or treated for anaemia within the Bangladesh health system.

The outcomes of interest were the percentage of pregnant women tested for anaemia in their previous pregnancy, diagnosed with anaemia who received treatment in their previous pregnancy, and received intravenous iron in any prior pregnancies. Percentages were only derived for women who were able to answer these questions (missing data and unsure responses were excluded from analysis).

Variables of interest included characteristics known to affect a woman’s access to and/or utilisation of health services (e.g., employment, education, decision-makers in healthcare),^34 43^ as well as those related to anaemia testing or treatment (e.g., testing location and type of treatments). Unsure responses for variables of interest were treated as a separate category and missing data were excluded from analysis. Households were categorised into five socioeconomic quintiles using a Wealth Index derived from principal components analysis of the household’s ownership of assets (e.g., television), housing characteristics (e.g., material of the roof), water source (e.g., piped water), and sanitation facilities (e.g., composting toilet) following the Demographic and Health Surveys (DHS) guidance for the construction of a Wealth Index.^44^

We undertook descriptive analyses and reported counts and percentages to explore the percentage of, and how, pregnant women were tested and treated for anaemia. To assess how the percentage of pregnant women tested for anaemia differed by social and equity dimensions (wealth quintile, women’s education, husband’s education, or person making decisions around woman’s healthcare), we derived unadjusted percentages and exact (Clopper-Pearson) 95% confidence intervals (CIs), stratified by the values of these dimensions. To visualise the data, we presented the results graphically using an equiplot (https://www.equidade.org/equiplot.php) created in R (V.4.2.2, R Foundation for Statistical Computing, Vienna, Austria).

To depict the different anaemia testing pathways for pregnant women in the Bangladesh health system, we created a Sankey diagram. Sankey diagrams are flow diagrams in which the nodes and links are proportional to the flow quantity.^45 46^ The Sankey diagram was created in Excel, using the ChartExpo Excel add-in (https://chartexpo.com/tools/excel). All other data analyses were conducted using Stata 17 software (StataCorp 2021. Stata Statistical Software: Release 17. College Station, Texas, USA: StataCorp LLC).

### Patient and public involvement

Patients and/or the public were not involved in the design, conduct, reporting, or dissemination plans of this research.

## Results

### Characteristics of women

Of the 1000 women who had previously been pregnant, 323 (32.3%) women were from Rupganj, 367 (36.7%) from Sonargaon, and 310 (31.0%) from Bandar upazila (table 1). Nearly all women were Muslim (954/997, 95.7%), followed by Hindu (43/997, 4.3%). Most women were aged between 20 and 24 years (394/1000, 39.4%) or 25 and 29 years (317/1000, 31.7%). The highest level of education most women had attained was primary education (524/997, 52.6%) or secondary education (395/997, 39.6%), with a minority attaining tertiary education (39/997, 3.9%), or no education (39/997, 3.9%). Most of the participating women’s husbands had a primary education (504/997, 50.6%) or secondary education (310/997, 31.1%). Almost all women were housewives (963/997, 96.6%), while 1.9% (19/997) had a manual job, and 1.0% (10/997) had a non-manual job. For 60.0% (600/1000) of women, decisions about their healthcare were made jointly with their husbands, and 7.2% (72/1000) of women made their own decisions about their healthcare. Women had a median monthly family income of 15 000 Bangladeshi Taka (IQR: 12 000–25 000, US$146 (conversion October 2021)).

**Table 1:** Characteristics of women (n=1000)

Variable	Frequency (%)
Residence (upazila)	
Rupganj	323 (32.3)
Sonargaon	367 (36.7)
Bandar	310 (31.0)
Woman’s religion*	
Islam	954 (95.7)
Hinduism	43 (4.3)
Woman’s age (years)	
15–19	68 (6.8)
20–24	394 (39.4)
25–29	317 (31.7)
30–34	178 (17.8)
35–39	41 (4.1)
40–45	2 (0.2)
Woman’s education level*	
No education	39 (3.9)
Primary education (1–8 years)	524 (52.6)
Secondary education (9–12 years)	395 (39.6)
Tertiary education (>12 years)	39 (3.9)
Husband’s education level*	
No education	129 (12.9)
Primary education (1–8 years)	504 (50.6)
Secondary education (9–12 years)	310 (31.1)
Tertiary education (>12 years)	54 (5.4)
Woman’s employment status*	
Housewife	963 (96.6)
Manual job	19 (1.9)
Non-manual job	10 (1.0)
Student	3 (0.3)
Other (probashi, tusoni)	2 (0.2)
Person making decisions about woman’s healthcare	
Woman and husband jointly	600 (60.0)
Husband	221 (22.1)
Other (mother-in-law, mother, father-in-law, father, brother, brother-in-law, sister-in-law, not specified)	107 (10.7)
Woman	72 (7.2)
Monthly family income (taka)†	
	15 000 (12 000–25 000)

* n=997. Manual job describes manual work (eg, garment worker, cook, cleaner). Non-manual job describes non-manual work (eg, administrative job, business owner, doctor). Probashi=lives abroad, Tusoni=tutor.

† n=996. Data presented as median (lower quartile–upper quartile) and in Bangladeshi Taka (US$1=103 Bangladeshi taka, conversion October 2021, Xe.com).

### Anaemia testing in pregnant women

**Figure 2:**
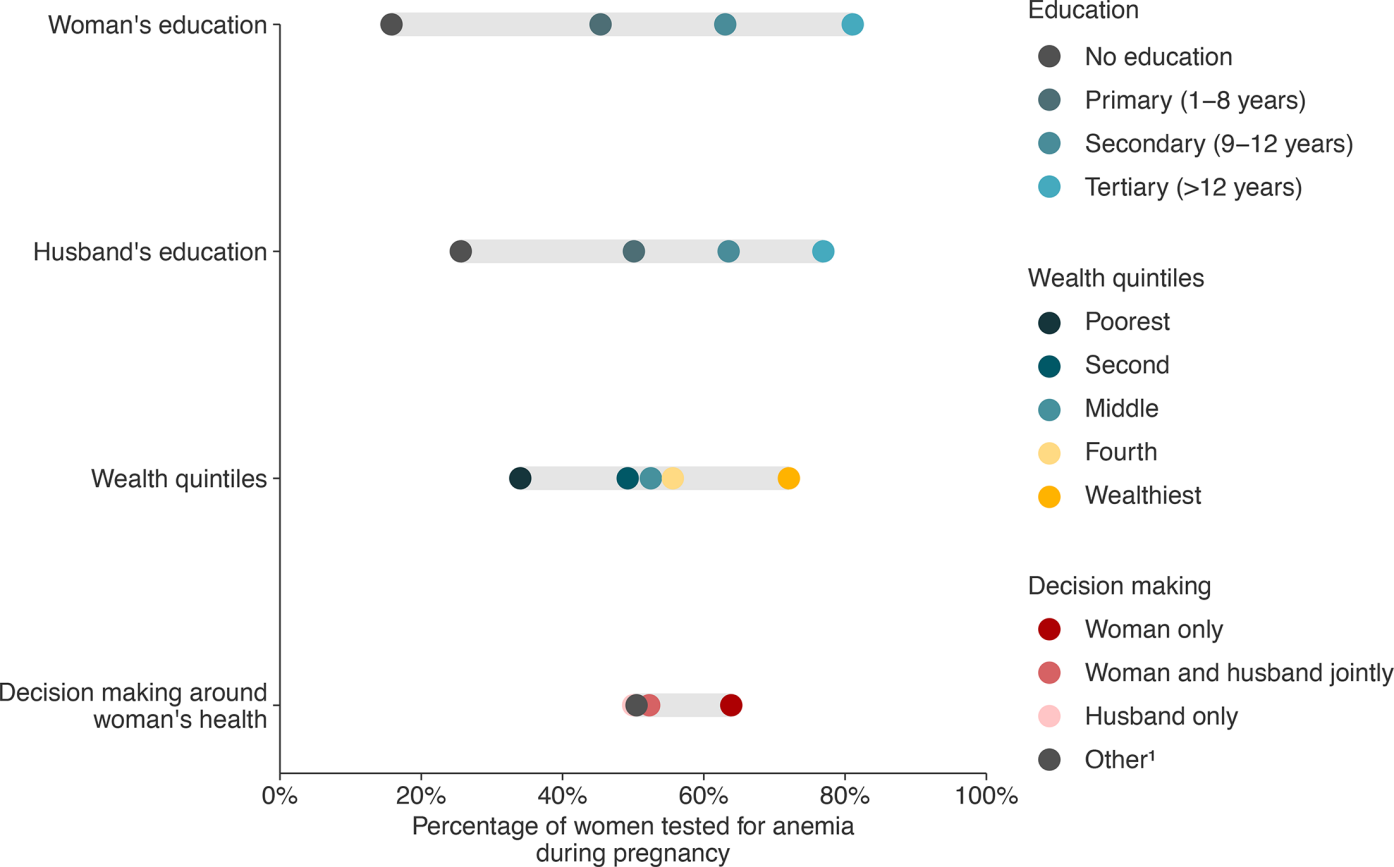
An equiplot of the percentage of pregnant women tested for anaemia by social and equity dimensions. ^1^Mother-in-law, mother, father-in-law, father, brother, brother-in-law, sister-in-law, not specified. In this equiplot, the dots represent the percentage of pregnant women tested for anaemia in each social and equity dimension (woman’s education, husband’s education, wealth quintiles, and decision-making around woman’s health), and the horizontal line shows the differences between values within each social and equity dimension. Estimates and their CIs are available in online supplemental table 2.

There were 963 women who could recall whether they were tested for anaemia in their previous pregnancy. Approximately half (505/963, 52.4%) of women reported that they were tested for anaemia (online supplemental table 1). The percentage of women tested for anaemia varied across social and equity dimensions (online supplemental table 2 and figure 2).

Women with no education were less likely to be tested for anaemia (15.8%, 95% CI: 6.0 to 31.3) compared to women with a primary education (45.4%, 95% CI: 41.0 to 49.8), secondary education (63.0%, 95% CI: 57.9 to 67.9), or tertiary education (81.1%, 95% CI: 64.8 to 92.0). Similarly, women whose husbands had no education were less likely to be tested for anaemia (25.6%, 95% CI: 18.2 to 34.2) compared to women whose husbands had a primary education (50.1%, 95% CI: 45.6 to 54.6), secondary education (63.5%, 95% CI: 57.7 to 69.0), or tertiary education (76.9%, 95% CI: 63.2 to 87.5).

Women from the poorest wealth quintile were less likely to be tested for anaemia (34.0%, 95% CI: 27.4 to 41.2) compared to women in the middle wealth quintile (52.5%, 95% CI: 45.3 to 59.6), and women in the wealthiest quintile (72.0%, 95% CI: 65.0 to 78.4).

Women who made their own decisions about their healthcare were more likely to be tested for anaemia (63.9%, 95% CI: 51.7 to 74.9) compared to women whose decisions about their healthcare were made jointly with their husband (52.3%, 95% CI: 48.1 to 56.4), by someone other (e.g., mother-in-law) (50.5%, 95% CI: 40.5 to 60.4), or by their husband only (50.0%, 95% CI: 43.1 to 56.9).

#### Place and methods of testing

**Figure 3:**
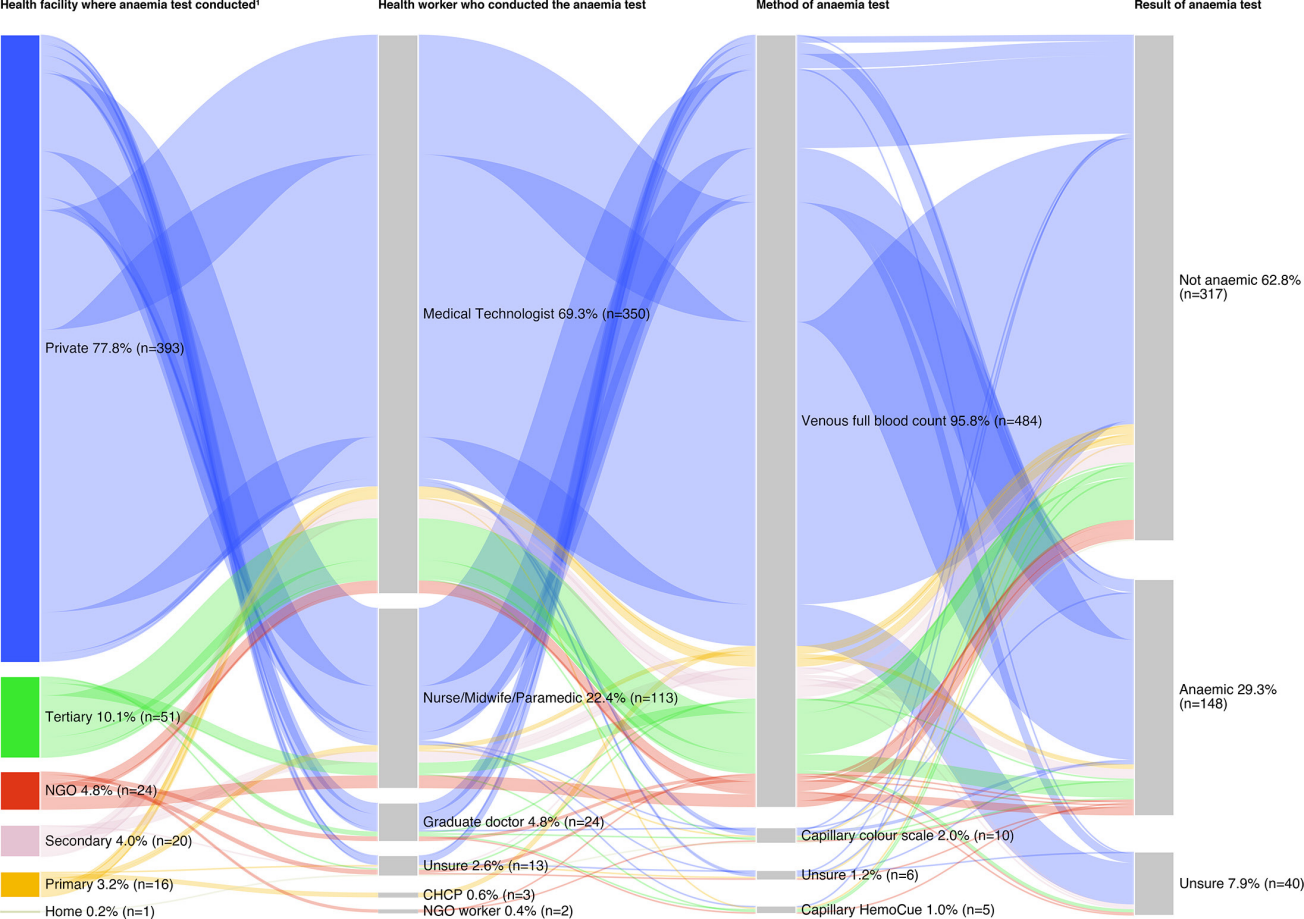
Sankey diagram of anaemia testing pathways for pregnant women in Bangladesh (n=505). ^1^Private health facilities (private practitioners chamber, private clinic/hospital, private medical college hospital). Primary health facilities (upazila health complex, union health and family welfare centre, satellite clinic, community clinic). Secondary health facilities (district/sadar hospital, mother and child welfare centre). Tertiary health facilities (public medical college hospital, tertiary hospital). The Sankey diagram visualises the percentages of women going through different aspects of anaemia testing (health facility where anaemia test conducted, health worker who conducted the anaemia test, method of anaemia test, and result of anaemia test). Colours identify different anaemia testing pathways for pregnant women in Bangladesh, with the size of the nodes and links proportional to the flow quantity. For example, the comparatively large width of the blue flow between private health facility, medical technologist, and venous full blood count shows that it is the most common anaemia testing pathway for pregnant women in Bangladesh. CHCP, community healthcare provider; NGO, non-governmental organisation.

Most of the 505 women who were tested for anaemia in their previous pregnancy were tested at a private facility (393/505, 77.8%), followed by a tertiary health facility (51/505, 10.1%) (online supplemental table 3). Over two-thirds of women were tested for anaemia by a medical technologist (350/505, 69.3%) and 22.4% (113/505) by a nurse, midwife, or paramedic. The majority of women were tested for anaemia by venous full blood count (484/505, 95.8%), followed by capillary colour scale (10/505, 2.0%), or capillary HemoCue (5/505, 1.0%). Following testing, 29.3% (148/505) of women were diagnosed as anaemic, 62.8% (317/505) as not anaemic, and 7.9% (40/505) were unsure of their diagnosis. Figure 3 visually depicts the anaemia testing pathways and shows that pregnant women in Bangladesh are most commonly tested at a private health facility, by a medical technologist, using venous full blood count.

### Treating anaemia in pregnant women

There were 142 women who could recall whether they received treatment following their anaemia diagnosis in their previous pregnancy. Most women received treatment (135/142, 95.1%), of which the majority received a single treatment (121/142, 85.2%) and 9.9% (14/142) multiple treatments (table 2).

**Table 2:** Treatment women received following anaemia diagnosis in their previous pregnancy (n=142) and intravenous iron use in any prior pregnancies (n=985)

Variable	Frequency (%)
Previous pregnancy	
Treatment women received following anaemia diagnosis*	
Single treatment	121/142 (85.2)
Multiple treatments	14/142 (9.9)
No treatment	7/142 (4.9)
Type of treatments for anaemia	
Iron and folic acid supplements only	99/135 (73.3)
Blood transfusion only	14/135 (10.4)
Intravenous iron only	8/135 (5.9)
Iron and folic acid supplements and blood transfusion	7/135 (5.2)
Iron and folic acid supplements and intravenous iron	7/135 (5.2)
Any prior pregnancies	
Women received intravenous iron†	
Yes	33/985 (3.4)
No	952/985 (96.7)
Number of infusions of intravenous iron women received in a single pregnancy	
1	11/33 (33.3)
2	14/33 (42.4)
3	3/33 (9.1)
4	2/33 (6.1)
5	3/33 (9.1)
Place where women received intravenous iron‡	
Private health facilities	14/33 (42.4)
Primary health facilities	8/33 (24.2)
Home	8/33 (24.2)
Secondary health facilities	2/33 (6.1)
Non-governmental organisation facilities	1/33 (3.0)

* Unsure (n=6) excluded from denominator.

† Unsure (n=15) excluded from denominator.

‡ Private health facilities (drug seller/store, private practitioners chamber, private clinic/hospital, private medical college hospital). Primary health facilities (upazila health complex, union health and family welfare centre, satellite clinic, community clinic). Secondary health facilities (district/sadar hospital).

Of the 135 women who were treated following their anaemia diagnosis, most received iron and folic acid tablets only (99/135, 73.3%), followed by blood transfusion only (14/135, 10.4%), intravenous iron only (8/135, 5.9%), iron and folic acid supplements and blood transfusion (7/135, 5.2%), and iron and folic acid supplements and intravenous iron (7/135, 5.2%).

### Intravenous iron use in pregnant women

To explore intravenous iron as a treatment option for pregnant women experiencing anaemia in Bangladesh, women were asked about intravenous iron use in any prior pregnancies. There were 985 women who could recall whether they received intravenous iron during prior pregnancies. Table 2 shows that a small number of these women received intravenous iron (33/985, 3.4%). In a single pregnancy, women reported that they had between one (11/33, 33.3%) and five infusions (3/33, 9.1%) of intravenous iron. Most women received intravenous iron at private health facilities (14/33, 42.4%), followed by at primary health facilities (8/33, 24.2%), or at home (8/33, 24.2%).

## Discussion

We describe how pregnant women are tested and treated for anaemia in Narayanganj district, Bangladesh, from analysis of 1000 women who participated in a cross-sectional survey. We found that approximately half of women were tested for anaemia in their previous pregnancy (505/963, 52.4%), with lower testing in women with no education (15.8%, 95% CI: 6.0 to 31.3), whose husband had no education (25.6%, 95% CI: 18.2 to 34.2), or in the poorest wealth quintile (34.0%, 95% CI: 27.4 to 41.2). We identified that pregnant women were most commonly tested for anaemia at a private health facility (393/505, 77.8%), by a medical technologist (350/505, 69.3%), using venous full blood count (484/505, 95.8%). Regarding treatment, we show that most women diagnosed with anaemia in their previous pregnancy received treatment (135/142, 95.1%). We found that in any prior pregnancies, a small number of women received intravenous iron (33/985, 3.4%), including in the primary health care system (8/33, 24.2%).

In our study, only about half of pregnant women were tested for anaemia, despite being part of WHO recommended routine antenatal care.^19^ This could be explained by health workers using clinical assessment alone (symptoms of fatigue and signs of conjunctiva and palmar pallor) to diagnose pregnant women with anaemia, rather than venous full blood count or capillary haemoglobinometer (clinical assessment is not recommended by WHO due to low accuracy).^19 47^ Further, we found that among the women tested for anaemia, most were tested using venous full blood count, which is the WHO recommended method for diagnosing anaemia in pregnancy.^19^ In contrast, only a small number of women were tested using the less resource-intensive point-of-care alternatives of capillary HemoCue and colour scale, which are recommended when venous full blood count is not available or feasible.^19^ This may be due to health workers in Bangladesh facing challenges in using point-of-care tests. Research conducted in other low- and middle-income countries has identified several factors affecting health workers’ ability to conduct point-of-care anaemia tests, including shortages of tests and associated equipment, inadequate training, concerns about the accuracy of results, and heavy workloads.^48^,^50^

Our equiplot (figure 2) highlighted that women with no education, whose husbands had no education, or from the poorest wealth quintile were less likely to be tested for anaemia in pregnancy. This finding aligns with a study conducted in Canada, which found that pregnant women in the lowest household income quintile had lower odds of being tested for iron deficiency compared to those in the highest income quintile.^51^ To the best of our knowledge, this is the first time inequities in anaemia testing rates among pregnant women have been demonstrated in Bangladesh. This is a concerning finding, particularly given previous research in Bangladesh showing that anaemia in women of reproductive age is more prevalent if the woman or her husband had no education, or if the woman was in the poorest wealth quintile.^52^ We found that most women who were tested and diagnosed with anaemia received treatment. Therefore, our findings demonstrate that the main issue is that only half of pregnant women are tested for anaemia, with substantial inequities in testing rates. As a result, pregnant women with anaemia—especially those who are least educated or poorest—are not receiving the treatment they need.

### Implications for research, including the EDIVA programme

Our study findings demonstrate the need for qualitative research with women and health workers to understand why women, particularly with no education, whose husband has no education, or in the poorest wealth quintile, are not being tested for anaemia in pregnancy.^53^ Further qualitative research should be conducted to identify why health workers are not using point-of-care tests such as capillary HemoCue, and why some pregnant women are not receiving treatment for anaemia. Our findings have provided substantial insights into the testing and treatment of anaemia in pregnant women in Bangladesh and may prompt similar research in other countries and settings where anaemia during pregnancy is also a major public health concern.

Our finding that some women received intravenous iron in pregnancy, including in the primary health care system, indicates the potential feasibility of the EDIVA programme to introduce it as a treatment option for moderate and severe anaemia. The EDIVA programme will provide further critical information required to formally implement, scale, and sustain an intravenous iron intervention.^54^ For example, a readiness assessment of primary health facilities will provide information on the availability of required equipment, resources, and medicines, and on the training health workers received to deliver the intervention.^55^ Qualitative interviews with women, health workers, and other key stakeholders will provide an understanding of the acceptability of an intravenous iron intervention delivered in the Bangladesh primary health care system.

### Implications for policy and practice

Our study reveals the need for substantial investments to ensure all women in Bangladesh are tested for anaemia in pregnancy. This could be achieved through increasing point-of-care testing in antenatal care. For example, the point-of-care capillary HemoCue does not require a functioning laboratory or medical technologist and could therefore be used across primary health facilities. Further, results from the HemoCue are immediate, which can improve women’s trust in the anaemia diagnosis and adherence to treatment.^49 56^ Improved testing may result in more women diagnosed with anaemia in pregnancy. To prepare for this, it would be integral that health facilities and health workers in Bangladesh can appropriately treat women. Overall, findings from our study may inform the design and implementation of strategies and programmes aimed at reducing the significant burden of anaemia among pregnant women in Bangladesh and other low- and middle-income countries.

### Strengths and limitations

To our knowledge, this is the first cross-sectional study to describe how women are tested and treated for anaemia in pregnancy in Bangladesh; and more broadly, in a setting where anaemia in pregnant women is a major public health concern. We conducted thorough descriptive analyses and reported our paper following STROBE guidelines.^37^ We are confident that our findings are representative of women across Narayanganj district, with women identified through government health facility registers. However, findings may not be generalisable to other districts, with the study site more urbanised than average and therefore likely to have a higher density of qualified health workers and private health facilities.^32^ While we were also interested in conducting an intersectionality analysis to explore how the percentage of women tested for anaemia differs by multiple forms of power and oppression, we were unable to because of the need for a very large and diverse sample.^57^,^59^ We acknowledge that our unidimensional measures do not capture the complexity of these women’s lives, or potential interactions between different social identities and conditions.^57^

### Conclusion

We found that only about half of women in Narayanganj district, Bangladesh, were tested for anaemia in their previous pregnancy, with lower testing in women with no education, whose husband had no education, or from the poorest wealth quintile. We also found that most women diagnosed with anaemia in their previous pregnancy received treatment, and a small number had received intravenous iron in prior pregnancies. Substantial investments are needed to ensure all women in Bangladesh are tested for anaemia in pregnancy and could include increasing point-of-care testing in antenatal care. However, health facilities and health workers may need to be prepared for an increase in the number of women diagnosed with anaemia. Overall, improving the testing and treatment of anaemia in pregnant women may contribute to reducing its’ significant burden in Bangladesh.

## Supplementary material

10.1136/bmjph-2024-002167 online supplemental file 1

10.1136/bmjph-2024-002167 online supplemental file 2

10.1136/bmjph-2024-002167 online supplemental file 3

10.1136/bmjph-2024-002167 online supplemental file 4

10.1136/bmjph-2024-002167 Abstract translation 1 This web only file has been produced by the BMJ Publishing Group from an electronic file supplied by the author(s) and has not been edited for content.

## Data Availability

Data are available upon reasonable request.
