# Supplementary material for: Testing and treating anaemia in pregnant women in Bangladesh: a cross-sectional survey
Source: BMJ Public Health. 2025 Jul 16;3(2):e002167. doi: 10.1136/bmjph-2024-002167 (PMC12273131; doi:10.1136/bmjph-2024-002167)
Supplement: online supplemental file 2 [file bmjph-3-2-s002.pdf]

Efficacy and Demonstration of IntraVenous Iron for Anaemia in pregnancy

(Activity1: Prevalence and Determinants of Anaemia in Bangladeshi Pregnant Woman.)

<u>অংশগ্রহনকারীর তথ্যঃ</u>

Screening ID:

Participant's name: Husband's Name:

Village:

Couple No: LMP:

Mobile no: FWA's name & unit:

## Section A: Eligibility Checking

**(সেকশন 'এ' ঃ গবেষণায় অন্তর্ভূক্তির যোগ্যতা যাচাইকরণ)**

১. সাক্ষাতের তারিখ ঃ |___|___|___|___| |___|___|___|__| সাক্ষাত গ্রহনকারীর নাম ঃ
Date of Visit Interviewer:

২. অংশগ্রহনকারীর সম্মতি প্রদানের তারিখ: |___|___|___|___| |___|___|___|__| 2.ক সম্মতি পত্রে স্বাক্ষর হয়েছে? |__| ১=হ্যাঁ,
Date of consent Is consent signed?
০=না, উত্তর যদি না হয়, তাহলে ২২ নং প্রশ্নে যান If No, skip to Q-22

৩. অংশগ্রহনকারীর নাম ঃ
Participant's Name

৪. অংশগ্রহনকারীর স্বামীর নাম ঃ
Participant's Husband Name

৫. এটি কার বাড়ী? ০ = স্বামীর বাড়ী ১ = শ্বশুড়ের বাড়ী (স্বামীর বাবা) ২ = বাবার বাড়ী (নিজের বাবা) , ৯৯= অন্যান্য,নির্দিষ্ট করুন -------
Whose house this is? 0=Husband's / 1=father in law's house (husband's father)/ 2=father's house, 99= Others, please specify..

৬. বাচ্চা প্রসবের সময় আপনি কোথায় থাকবেন বলে পরিকল্পনা করেছেন?
What is your plan to stay during delivery?
০ = স্বামীর বাড়ী ১ = শ্বশুড়ের বাড়ী (স্বামীর বাবা) ২ = বাবার বাড়ী (নিজের বাবা), ৯৯= অন্যান্য, নির্দিষ্ট করুন------
0=Husband's / 1=father in law's house (husband's father)/ 2=father's house, 99= Others, please specify..

৭. প্রসবের সময় আপনি কোন এলাকায় থাকবেন বলে ঠিক করেছেন?
Where is the place you have selected for delivery?

১ = রূপগঞ্জ, ২ = সোনারগাঁ, ৩ = বন্দর ৪ = অন্যান্য: উপজেলা. ........................................

1= Rupganj, 2=Sonargaon, 3= Bandar, 4= Other; Upazila

৮. খানা প্রধানের নাম ঃ ........................................... /____/
Head of the Household Name
(০= নিজেই, ১=স্বামী, ২= শ্বশুর, ৩= নিজের বাবা, ৯৯=অন্যান্য অভিভাবক/ উল্লেখ করুন---)
(0= Self, 1=Husband, 2=Father-in-law 3=Father, 99=Other guardian /specify_____)

৯. বাড়ীর নাম ঃ
Bari Name

১০. গ্রামের নাম ঃ
Village Name

১১. ইউনিয়ন :

Union Name

১২. উপজেলার নামঃ-----------------/---/, ১ = রূপগঞ্জ, ২ = সোনারগাঁ, ৩ = বন্দও, ৪ = অন্যান্যঃ উপজেলা. ----------

Upazila Name 1= Rupganj, 2=Sonargaon, 3= Bandar, 4= Other; Upazila

১৩. আপনার (অংশগ্রহনকারীর) মোবাইল নম্বর ঃ |__|__|__|__|__|___|___|___|__|___|___|__|

Participant's mobile number

১৩.ক. অন্যান্যঃ |__|__|__|__|__|___|___|___|__|___|___|__| , সম্পর্ক /__/ ১=স্বামী, ২= শ্বশুর, ৩= নিজের বাবা,৪= আত্মীয়

Other ৫= প্রতিবেশী (1=Husband, 2=Father-in-law, 3=Father, 4=Relative, 5=Neighbour 6=Other; specify)

১৪. আপনার বয়স ঃ /___/___/ বছর

Age:

১৫. শেষ মাসিকের তারিখ (এল,এম, পি) (FWA রেজিষ্টার অনুসারে): |___|___|___|___| |___|___|___|__| দিন/মাস/বছর

Date of Last Menstrual Period (LMP) (According to FWA register):

১৫.ক. সংশোধিত এল,এম, পি |___|___|___|___| |___|___|___|__| দিন/মাস/বছর ,

Corrected LMP

১৫.খ. সংশোধনের কারনঃ /__/, ১= FWA রেজিষ্টারে ভুল এন্ট্রি, ২= আলট্রাসাউন্ড রিপোর্টের সাথে তারিখের বড়অমিল, ৩=অন্যান্য,

Reason for correction বর্ণনা করুন----- 1= Wrong entry in FWA register, 2= Date Mismatch with USG report, 3= Others, specify

১৬. আনুমানিক গর্ভকালীন বয়স ঃ /___/___/ সপ্তাহ [autofill from Qes 15, LMP+277]

Estimated Gestational Age:

১৭. আনুমানিক প্রসবের তারিখ ঃ |___|___|___|___| |___|___|___|__| দিন/মাস/বছর

Estimated date of delivery:

১৮. **গবেষণায় অন্তর্ভূক্তি হওয়ার বৈশিষ্ট্য সমুহঃ**

**Eligibility criteria:**

১৯. FWA রেজিষ্টার অনুসারে ১৩ - ৩২ সপ্তাহের গর্ভবতী : /___/ ০ = না ১ = হ্যাঁ

13-32 weeks gestation by FWA register 0=No, 1=Yes

২০. গবেষণার আওতাধীন এলাকার (রূপগঞ্জ/ সোনারগাঁ/ বন্দর) বাসিন্দা ০ = না , ১ = হ্যাঁ /___/

Resident in the study catchment area 0=No, 1=Yes

২১. অংশগ্রহনকারী সম্মতিদানে আগ্রহী ০ = না, ১ = হ্যাঁ /___/

Participant agree to give consent 0=No, 1=Yes

২২. সম্মতি না দেওয়ার কারন কি? ১ = আমি আগ্রহী নই ২ = স্বামী/পরিবারের অসম্মতি ৩ = শিরা হতে রক্ত দিতে অসম্মতি ৪ = অন্যান্য---

What is the reason for denial consent? /___/ 1=I am not interested, 2= Husband/Mother-in-law's disapproval, 3= Venous Blood refusal, 4=Other, Specify______

২৩. গবেষণা জরীপ/সার্ভের জন্য সে কি যোগ্য? **(১৯, ২০ ও ২১ নং প্রশ্নের সব উত্তর হ্যাঁ হলে উত্তরপ্রদানকারী গবেষণায় অংশগ্রহনের জন্য যোগ্য)**

Is she eligible for the survey ০ = না (সাক্ষাতকার শেষ করুন) ১ = হ্যাঁ /___/ 0=No [Stop interview], 1=Yes

*[ নির্দেশনা : ২৩ নং প্রশ্নের উত্তর হ্যা হলে উত্তরপ্রদানকারীকে গবেষনায় অংশগ্রহনের পরবর্তী ধাপ বর্ণনা করুন]*

## Section B: Physical Examination

*This time I would like to check your anaemia, blood pressure, pulse rate and measure height -weight and MUAC*

| | | |
|---|---|---|
| 1 | Actual time of measurement (in 24hrs format) | : [auto-fill] |
| 2 | Sign of pallor | \|___\| 0=No,1=Mild, 2=Moderate, 3=Severe |
| 3 | Pulse Rate | \|___\|___\|___\| beats/minute |
| 4 | Presence of oedema | \|___\| 0=No 1= Yes |
| 5 | Sitting position Blood Pressure | ____________/___________ mmHg |
| | | Systolic Diastolic |
| 6 | Weight 1 | \|___\|___\|___\|.\|___\| Kg |
| | Weight 2 | \|___\|___\|___\|.\|___\| Kg |
| 7 | Height 1 | \|__\|__\|__\| . \|__\| cm |
| | Height 2 | \|__\|__\|__\|.\|__\| cm |
| 8 | Mid upper-arm circumference measurement | \|__\|__\| . \|__\| cm |

## <u>Section C : Socio-Demographics</u>

**(সেকশন 'সি' ঃ সামাজিক জন-তাত্বিক তথ্য)**

[এই সেকশনে আমি আপনাকে আপনার আপনার আর্থ-সামাজিক অবস্থা সম্পর্কে জানতে চাইব। কোথাও কিছু বুঝতে না পারলে অনুগ্রহপূর্বক আমাকে খোলাখুলিভাবে জানাবেন।]

১. আপনি কি কখনো প্রচলিত স্কুলে পড়েছেন? ০ = না **(উত্তর "না" হলে ৩নং প্রশ্নে যান)** ১ = হ্যাঁ /___/ *0= No (**if no skip to Q03**) 1= Yes*

Have you ever attended formal school?

২. আপনি কতদূর পর্যন্ত পড়েছেন?

How far did you go with your education?*(Choose one)*

শ্রেনী (I- V) /___/ শিক্ষা সমাপ্তির পূর্ণ বছর /___//___/ বছর

*Class (I-V),* |__| |__|__| *years*

শ্রেনী (VI- X) /___/ শিক্ষার সমাপ্তির পূর্ণ বছর /___//___/ বছর

*Class (VI-X),* |__| |__|__| *years*

এস,এস,সি/দাখিল /___/ শিক্ষার সমাপ্তির পূর্ণ বছর /___//___/ বছর

*SSC/Dakhil,* |__| |__|__| *years*

এইচ,এস,সি/আলিম /___/ শিক্ষার সমাপ্তির পূর্ণ বছর /___//___/ বছর

*HSC/Alim,* |__| |__|__| *years*

স্নাতক/ফাজিল /___/ শিক্ষার সমাপ্তির পূর্ণ বছর /___//___/ বছর

*Graduate/Fazil* |__| |__|__| *years*

স্নাতকোত্তর/কামিল /___/ শিক্ষার সমাপ্তির পূর্ণ বছর /___//___/ বছর

*Post-graduate/Kamil*|__| |__|__| *years*

৩. আপনি কি পড়তে পারেন? ০ = না, ১ = হ্যাঁ /___/

Are you able to read? *0= No 1= Yes*

৪. আপনি কি লিখতে পাড়েন? ০ = না, ১ = হ্যাঁ /___/

Are you able to write? *0= No 1= Yes*

৫. আপনি কোন ধর্ম পালন করেন? (যে কোন একটি বাছাই করুন) ০ = ইসলাম, ১ = হিন্দু, ২ = খ্রিষ্টান, ৩ = বৌদ্ধ, ৪= অন্যান্য ঃ উল্লেখ করুন ........ /___/

What religion do you practice?*(Choose one)* *0= Islam, 1= Hinduism, 2= Christian, 3= Buddhist, 99= Other, specify…..*

৬. আপনার প্রধান পেশা কি? (যে কোন একটি বাছাই করুন) /___/

What is your main employment?*(Choose one)*

০ = বেকার

*Unemployed*

১ = ছাত্র/ছাত্রী (চাকুরীরত নয়)

*Student (not employed)*

২ = গৃহিনী/গৃহস্থালী কাজকর্ম ( চাকুরীরত নয়)

*Housewife/ household activities (not employed)*

৩ = ভিক্ষুক (চাকুরীরত নয়)

*Beggars(not employed)*

৪ = প্রতিবন্ধী (চাকুরীরত নয়)

*Disabled (not employed)*

৫ = অবসরপ্রাপ্ত/পেনশনার (চাকুরীরত নয়)

*Retired/ Pensioners (not employed)*

৬ = অদক্ষ (দৈহিক/কায়িক শ্রম) ঃ গৃহপরিচারিকা, আয়া, গার্মেন্টস শ্রমিক (পরিস্কার পরিচ্ছন্ন,খাবার বিতরন,নিরাপত্তার কাজে নিযুক্ত গার্মেন্টস কর্মী), উৎপাদন/নির্মান কর্মী, বাস/টেম্পু হেলপার, গার্ড/দারোয়ান.পরিচ্ছন্ন কর্মী, পিওন/অনুচর, রিক্সা/ভ্যান/ঠেলা গাড়ী চালক, দিন মজুর, কৃষক, জেলে।

*Manual Unskilled: Housemaid, aya, garment worker (garments worker involved in cleaning, food distribution, security), production/ construction worker, bus/tempo helper, guard/darowan, cleaner, peon/attendant, rickshaw/van/pushcart puller, day labourer, farmer, fisherman*

৭ = দক্ষ (দৈহিক/ কায়িক শ্রম) ঃ গার্মেন্টস কমী (জামা কাপড় তৈরী বা উৎপাদনে নিযুক্ত), রাধুঁনি, উৎপাদন/ নির্মানকর্মী, পরিবহন চালক

*Manual Skilled: garment worker (involved in making dress or production), cook, production/ construction worker, transport driver*

৮ = অন্যান্য দক্ষ (দৈহিক শ্রম/ কায়িক) ঃ দর্জি, নাপিত, কারিগর, ছুতার/কাঠ মিস্ত্রী,মোটরগাড়ি কর্মী,পেট্রোল পাম্প কর্মী, চিত্রশিল্পী, মুচি।

*Other manual skilled: Tailor, barber, craftsman, carpenter, automobile worker, petrol pump worker, painter, cobbler*

৯ = (দৈহিক /কায়িক শ্রম নয়) ঃ সরকারী, বেসরকারী, গার্মেন্টস বা ব্যাক্তি মালিকানাধীন প্রতিষ্ঠানের প্রশাসনিক/করণিক চাকুরী, সরকারী/বেসরকারী প্রতিষ্ঠানের আওতাধীন কমী, বিক্রয় কর্মী।

*(Non-manual) Administrative/ clerical jobs in GO, NGO, garment or private organizations, GO/NGO extension workers, salesperson*

১০ = (দৈহিক / কায়িক শ্রম নয়) ঃ প্রথম সারীর স্বাস্থ্যসেবা প্রদানকারী, গ্রাম্য চিকিৎসক, হোমিওপ্যাথ, কবিরাজ, প্রচলিত ধাত্রী, ফার্মাসিষ্ট, সরকারী/ বেসরকারী কমিউনিটি স্বাস্থ্য কর্মী।

*(Non-manual) First line health service providers: village doctors, homepaths, kabiraj, TBA, pharmacist, GO/NGO community health workers*

১১ = (দৈহিক / কায়িক শ্রম নয়) ঃ বণিক, নিজস্ব ব্যবসা

*(Non-manual) Traders, business owners*

১২ = (দৈহিক / কায়িক শ্রম নয়) পেশাদারী ঃ ডাক্তার, কৃষিবিদ, শিক্ষক, ইঞ্জিনিয়ার

*(Non-manual) Professional: Doctors, agriculturist, teacher, engineer*

১৩ = অজানা

*Unknown*

৯৯ = অন্যান্য ঃ উল্লেখ করুন .....................................

*Other; specify*

৭. অংশগ্রহনকারীর স্বামীর বয়সঃ /___/___/ বছর

Participnat's Husband Age in years |__|__|__| *years*

৮. আপনার স্বামী কি কখনো প্রচলিত স্কুলে পড়েছেন? ০ = না **(উত্তর "না" হলে ১০ নং প্রশ্নে যান)** ১ = হ্যাঁ /___/

Has your husband ever attended formal school? 0= No (if no skip to Q10),1=Yes

৯. তিনি কতদূর পর্যন্ত পড়েছেন? (যে কোন একটি বাছাই করুন)

How far did he go with your education?

শ্রেনী (I- V) /___/ শিক্ষা সমাপ্তির পূর্ণ বছর /___//___/ বছর

*Class (I-V),* |__| |__|__| *years*

শ্রেনী (VI- X) /___/ শিক্ষার সমাপ্তির পূর্ণ বছর /___//___/ বছর

*Class (VI-X),* |__| |__|__| *years*

এস,এস,সি/দাখিল /___/ শিক্ষার সমাপ্তির পূর্ণ বছর /___//___/ বছর

*SSC/Dakhil,* |__| |__|__| *years*

এইচ,এস,সি/আলিম /___/ শিক্ষার সমাপ্তির পূর্ণ বছর /___//___/ বছর

*HSC/Alim,* |__| |__|__| *years*

স্নাতক/ফাজিল /___/ শিক্ষার সমাপ্তির পূর্ণ বছর /___//___/ বছর

*Graduate/Fazil* |__| |__|__| *years*

স্নাতকোত্তর/কামিল /___/ শিক্ষার সমাপ্তির পূর্ণ বছর /___//___/ বছর

*Post-graduate/Kamil*|__| |__|__| *years*

১০. তিনি কি পড়তে পারেন? ০ = না, ১ = হ্যাঁ /___/

Is he able to read? *0= No 1= Yes*

১১. তিনি কি লিখতে পাড়েন? ০ = না, ১ = হ্যাঁ /___/

Is he able to write? *0= No 1= Yes*

১২. আপনার স্বামীর প্রধান পেশা কি? (যে কোন একটি বাছাই করুন) /___/

What is your husband's main employment? *(Choose one)*

০ = বেকার

*Unemployed*

১ = ছাত্র/ছাত্রী (চাকুরীরত নয়)

*Student (not employed)*

২ = গৃহিনী/গৃহস্থালী কাজকর্ম ( চাকুরীরত নয়)

*Housewife/ household activities (not employed)*

৩ = ভিক্ষুক (চাকুরীরত নয়)

*Beggars(not employed)*

৪ = প্রতিবন্ধী (চাকুরীরত নয়)

*Disabled (not employed)*

৫ = অবসরপ্রাপ্ত/পেনশনার (চাকুরীরত নয়)

*Retired/ Pensioners (not employed)*

৬ = অদক্ষ (দৈহিক/কায়িক শ্রম) ঃ গৃহপরিচারিকা, আয়া, গার্মেন্টস শ্রমিক (পরিস্কার পরিচ্ছন্ন,খাবার বিতরন,নিরাপত্তার কাজে নিযুক্ত গার্মেন্টস কর্মী), উৎপাদন/নির্মান কর্মী, বাস/টেম্পু হেলপার, গার্ড/দারোয়ান.পরিচ্ছন্ন কর্মী, পিওন/অনুচর, রিক্সা/ভ্যান/ঠেলা গাড়ী চালক, দিন মজুর, কৃষক, জেলে।

*Manual Unskilled: Housemaid, aya, garment worker (garments worker involved in cleaning, food distribution, security), production/ construction worker, bus/tempo helper, guard/darowan, cleaner, peon/attendant, rickshaw/van/pushcart puller, day labourer, farmer, fisherman*

৭ = দক্ষ (দৈহিক/ কায়িক শ্রম) ঃ গার্মেন্টস কমী (জামা কাপড় তৈরী বা উৎপাদনে নিযুক্ত), রাধুঁনি, উৎপাদন/ নির্মানকর্মী, পরিবহন চালক

*Manual Skilled: garment worker (involved in making dress or production), cook, production/ construction worker, transport driver*

৮ = অন্যান্য দক্ষ (দৈহিক শ্রম/ কায়িক) ঃ দর্জি, নাপিত, কারিগর, ছুতার/কাঠ মিস্ত্রী,মোটরগাড়ি কর্মী,পেট্রোল পাম্প কর্মী, চিত্রশিল্পী, মুচি।

*Other manual skilled: Tailor, barber, craftsman, carpenter, automobile worker, petrol pump worker, painter, cobbler*

৯ = (দৈহিক /কায়িক শ্রম নয়) ঃ সরকারী, বেসরকারী, গার্মেন্টস বা ব্যাক্তি মালিকানাধীন প্রতিষ্ঠানের প্রশাসনিক/করণিক চাকুরী, সরকারী/বেসরকারী প্রতিষ্ঠানের আওতাধীন কমী, বিক্রয় কর্মী।

*(Non-manual) Administrative/ clerical jobs in GO, NGO, garment or private organizations, GO/NGO extension workers, salesperson*

১০ = (দৈহিক / কায়িক শ্রম নয়) ঃ প্রথম সারীর স্বাস্থ্যসেবা প্রদানকারী, গ্রাম্য চিকিৎসক, হোমিওপ্যাথ, কবিরাজ, প্রচলিত ধাত্রী, ফার্মাসিষ্ট, সরকারী/ বেসরকারী কমিউনিটি স্বাস্থ্য কর্মী।

*(Non-manual) First line health service providers: village doctors, homepaths, kabiraj, TBA, pharmacist, GO/NGO community health workers*

১১ = (দৈহিক / কায়িক শ্রম নয়) ঃ বণিক, নিজস্ব ব্যবসা

*(Non-manual) Traders, business owners*

১২ = (দৈহিক / কায়িক শ্রম নয়) পেশাদারী ঃ ডাক্তার, কৃষিবিদ, শিক্ষক, ইঞ্জিনিয়ার

*(Non-manual) Professional: Doctors, agriculturist, teacher, engineer*

১৩ = অজানা

*Unknown*

৯৯ = অন্যান্য ঃ উল্লেখ করুন ....................................

*Other; specify*

১৩. আপনি ও আপনার স্বামীর মাসিক আয় কত (টাকায়)?------------------

How much do you and your husband earn together per month? (in Taka)

১৪. আপনার পরিবারের (খানায়) মাসিক আয় কত? (টাকায়) [সকল সদস্যের আয় মিলিয়ে]ঃ ১) .............................টাকা, ২) জানিনা...........

How much is your monthly family income? (in Taka) [From all sources] 1=|__|__|__|__|__|__|, 2= Unknown

১৫. আপনার পরিবারের মাসিক খরচ কত? (টাকায়) ঃ ...................................

How much is your monthly family expenditure? (in Taka)

১৬. মাসিক আয় ও ব্যায়ের ঘাটতি ঃ /___/

Deficit between monthly family income & deficit (in Taka)

০ = সব সময় ঘাটতি থাকে, ১ = মাঝে মাঝে ঘাটতি থাকে, ২= সমান সমান ৩ = উদ্বৃত থাকে ৪=অন্যান্য (উল্লেখ করুন) ...................................

*0= Deficit always present, 1= Deficit occasionally present, 2= Balance, 3= Surplus, 4= Other, specify.....*

১৭. বসত বাড়ির অবস্থা ঃ ১ = ভাড়া বাড়ি, ২= নিজের বাড়ি, ৩ = অন্যান্য (উল্লেখ করুন).................................. /___/

Household status: 1=rented house, 2= owned house, 99=other (please specify)

১৮. বাড়ির ছাদ কিসের তৈরী? ১ = শুকনো পাতা বা খড়, বাঁশ, ২ = টিন, ৩ = সিমেন্ট, ৪ = অন্যান্য (উল্লেখ করুন)...............................

The roof of the house is made up of: 1=dry leaves or straw, bamboo, 2=tin, 3=Cement, 99=other (please specify…)

১৯. বাড়ির মেঝে কিসের তৈরী? ১ = কাঁদা /বাঁশ, ২ = কাঠ, ৩ = সিমেন্ট, ৪ = অন্যান্য (উল্লেখ করুন) ...............................

The floor of the house is made up of : 1=mud/ bamboo, 2=wood, 3=cement, 99= other (please specify…)

২০. বাড়ির দেয়াল কিসের তৈরী? ১ = শুকনো পাতা বা খড়, বাঁশ, ২ = কাদা, ৩ = টিন, ৪ = সিমেন্ট, ৭ = অন্যান্য (উল্লেখ করুন) .................

The wall of the house is made up of: 1= dry leaves or straw, bamboo,2=mud, 3=tin,4=cement,99= other (mention specifically)

২১. আপনার পরিবারের (খানায়)সদস্য সংখ্যা কয়জন? /_____/

Total number of family members in your Household (including yourself)?

২২. বসবাসের জন্য আপনার বাড়িতে (খানায়) কয়টা ঘর আছে? /_____/

Total number of rooms in your house for living?

২৩. আপনার নিজের জমি আছে? (বসবাসের জায়গা ছাড়া) ১ = হ্যাঁ , ০ = না /___/

Do you have any land of your own (except land used for house)? 1= Yes 0= No

২৪. আপনার বাড়িতে নিচের কোন জিনিসগুলো আছে? (একাধিক উত্তর গ্রহনযোগ্য) ১ = হ্যাঁ , ০ = না /___/

Which of the following items do you have in your house? 1= Yes 0= No

a) ড্রেসিং টেবিল /__/ b) চেয়ার /__/, c ) টেলিভিশন /__/, d) কম্পিউটার /__/, e) মহিষ/গরু /__/, f) মোবাইল /__/,

g) ফ্যান /__/, h) আলনা, i) ফ্রিজ /__/, j) হাঁস.মুরগী /__/

a. Dressing table, b. Chair c. Television d. Computer / Laptop e. Buffalo/Cow f. Mobile g. Fan h. Stand for cloths i. Refrigerator j. Duck/Hen

২৫. আপনার বাড়িতে বৈদ্যতিক সংযোগ আছে? ১ = হ্যাঁ, ০ = না /___/

Do you have electricity connection in your house? 1=Yes, 0= No

২৬. আপনি রান্নার জন্য কোন জ্বালানী ব্যবহার করেন (সচরাচর ব্যবহৃত)? /_____/

What type of fuel do you use for cooking (frequently used)?

৯ = ইলেকট্রিসিটি, ৮ = এলপিজি, ৭ = প্রাকৃতিক গ্যাস, ৬ = বায়ো গ্যাস, ৫ = কেরোসিন, ৪= কয়লা/ কয়লা জাতীয় বস্তু/কাঠের কয়লা,

৩ = কাঠ, ২ = লাকড়ী/ লতা-পাতা/ জমির ফসল, ১ = পশুর গোবর, ৯৯ = অন্যান্য (উল্লেখ করুন) .................

Electricity=9, LPG=8, Natural Gas=7, Bio-Gas=6, Kerosene=5, Coal/Coal like matter/wooden coal=4, Wood=3,

Straw/Grass/Agricultural crop=2, Animal dung=1, 99= Other (please specify....)

২৭. রান্না সাধারনতঃ কোথায় করা হয়? /____/

Where do you cook in your house?

১ = শোবার ঘরের মধ্যেই, ২=শোবার ঘরের সাথেই ৩ = আলাদা স্থাপনায়, ৪ = বাহিরে খোলা জায়গায়, ৫ = অন্যান্য (উল্লেখ করুন) ....................

1=In the bed room, 2=Attached to the bed room, 3=Separate building, 4= Open outdoors spaces, 5=Other, specify……..

২৮. আপনি কি ধূমপান করেন বা তামাক/জর্দ্দা সেবন করেন? ০ = না /___/, ১ = হ্যাঁ, ধূমপান করেন, তামাক /___/ জর্দ্দা /___/

Do you smoke or take smokeless tobacco? 0=No, 1=Yes; Smoke |_|, Smokeless tobacco|__|

২৯. আপনার পরিবারে কেউ ধূমপান করেন? ০ = না /___/, ১ = হ্যাঁ, স্বামী /___/, ২ = হ্যাঁ, অন্যকেউ, (উল্লেখ করুন) /___/

Does anyone in your household smoke? 0=No, 1=Yes, husband, 2=Yes, other_____

| | | |
|---|---|---|
| | **Section D: Pregnancy history** | |
| 1 | How many pregnancies have you had including this one (gravidity)? **(If first pregnancy, Response=1 skip to Q19)**<br>আপনি মোট কতবার গর্ভবতী হয়েছেন (এইবার সহ)? | *Number:*\|___\|___\| |
| 2 | Total number of live births (Born live after 28 weeks of gestation)<br>কতবার জীবিত সন্তান প্রসব করেছেন (parity: ২৮ সপ্তাহের পরে জীবিত বাচ্চা প্রসবের সংখ্যা**)** | *Number:*\|___\|___\| |
| 3 | Number of still births (Born death after 28 weeks of gestation)<br>মৃত বাচ্চা প্রসবের সংখ্যা? (২৮ সপ্তাহের পরে মৃত বাচ্চা প্রসবের সংখা সংখ্যা) | *Number:*\|___\|___\| |
| 4 | Number of abortions/miscarriages (≤ 28 weeks of gestation)<br>গর্ভপাত (২৮ সপ্তাহ বা তার পূর্বে গর্ভপাত) মোট কতবার হয়েছে? | *Number:*\|___\|___\| |
| 5 | Number of vaginal deliveries<br>স্বাভাবিক প্রসবের (vaginal delivery) সংখ্যা | *Number:*\|___\|___\| |
| 6 | Number of Caesarean sections<br>সিজারিয়ান সেকশনের সংখ্যা | *Number:*\|___\|___\| |
| 7 | How old were you at the time of your first pregnancy?<br>আপনি যখন প্রথমবার গর্ভবতী হোন তখন আপনার বয়স কত ছিল? | \|__\|__\| *years* |
| 8 | Status of your last pregnancy outcome?<br>আপনার সর্বশেষ গর্ভের ফলাফল কি?<br>1= live birth, 2= still birth, 3= abortion | \|___\| |
| 9 | Where did the last pregnancy outcome take place? *(Choose one)*<br>আপনার সর্বশেষ ডেলিভারি/গর্ভপাত কোথায় হয়েছে? | \|___\| |
| | 0=Private practitioner’s chamber,<br>1= Private facilities (clinics/hospitals) | |

| | | |
|---|---|---|
| | 2= Public medical college hospital<br>3= Private medical college hospital<br>4= Tertiary hospital<br>5=District Hospital/Sadar hospital<br>6=Upazila Health Complex (UHC)<br>7= Union Sub-center<br>8= Union Health & Family Welfare Center (UH& FWC)<br>9= Satellite clinic<br>10=Community Clinic<br>11= Maternal and Child Welfare Centre<br>12= NGO facilities<br>13= On the way to hospital/health center<br>14= Own home<br>15= Other's home<br>99=Other, Specify______ | |
| 10 | Who was helping you at the time of last birth or pregnancy outcome? *(Choose one)*<br>বাচ্চা প্রসব বা ডেলিভারির সময় আপনাকে কে সহায়তা করেছিল? | \|___\|__\| |
| | 1= Graduate doctor<br>2= Nurse/Midwife/Paramedic<br>3= Family Welfare Visitor (FWV)<br>4= Sub-Assistant Community Medical Officer (SACMO)<br>5= Community Health Care Provider (CHCP)<br>6= Family Welfare Assistant (FWA)<br>7= Health Assistant (HA)<br>8= NGO worker<br>9= Trained Traditional Birth Attendant (TBA)<br>10= UntrainedTraditional Birth Attendant (UTBA)<br>11= Unqualified doctor<br>12= Relative/Neighbour<br>13= No one assisted<br>14= Unsure<br>99=Other, Specify______ | |
| 11 | Can you tell me the gap between current and last pregnancy?<br>আপনার বর্তমান ও পূর্বের গর্ভাবস্থার মধ্যে সময়ের পার্থক্য কত? | \|___\|__\| *মাস* |
| | **History of anaemia and iron supplement used in previous pregnancy** | |
| 12 | Were you tested for anaemia in any previous pregnancy? (By checking blood haemoglobin)<br>আপনার আগের কোন গর্ভকালীন সময়ে রক্তের হিমোগ্লোবিন থেকে রক্ত-স্বল্পতা পরীক্ষা করা হয়েছে কি? | \|__\| |
| | | 0 = No (Skip to Q17 a )<br>1= Yes<br>2= Unsure (Skip to Q17 a) |
| 13 | Where was the anaemia test done (Choose one)<br>কোথায় রক্ত-স্বল্পতা পরীক্ষা করা হয়েছিল? | \|__\|__\| |
| | 0= Drug seller/store<br>1=Private practitioner's Chamber,<br>2= Private facilities (clinics/hospitals)<br>3= Public medical college hospital<br>4= Private medical college hospital<br>5= Tertiary hospital<br>6=District Hospital/sadar hospital<br>7=Upazila Health Complex (UHC)<br>8= Union Sub-center<br>9= Union Health & Family Welfare Center (UH& FWC) | |

| | | |
|---|---|---|
| | 10= Satellite clinic<br>11=Community Clinics<br>12= Maternal and Child Welfare Centre<br>13= NGO facilities<br>14= At home<br>99=Other, Specify______ | |
| 14 | Who performed the anaemia test?<br>কে রক্ত-স্বল্পতা পরীক্ষা করেছিল? | \|__\|__\| |
| | 1= Graduate doctor<br>2= Nurse/Midwife/Paramedic<br>3= Medical Technologist<br>4= Family Welfare Visitor (FWV)<br>5= Sub-Assistant Community Medical Officer (SACMO)<br>6= Community Health Care Provider (CHCP)<br>7= Family Welfare Assistant (FWA)<br>8= Health Assistant (HA)<br>9= NGO worker<br>10= Unqualified doctor<br>11= Unsure<br>99=Other, Specify______ | |
| 15 | How was the anaemia test done?<br>কিভাবে রক্ত-স্বল্পতা পরীক্ষা করেছিল? | \|__\| |
| | 0= Finger prick HemoCue<br>1= Finger prick Hemocheck (colour scales)<br>2= From venous blood<br>3= Unsure | |
| 16 | What was the result of the test?<br>পরীক্ষার ফলাফল/ রেজাল্ট কি ছিল? | 0= Not anaemic (Skip to Q17 a )<br>1=Anaemic<br>2= Unsure (Skip to Q17 a ) |
| 17 | What treatment was provided for anaemia? Multiple response allowed<br>সে সময়ে কি চিকিৎসা দেওয়া হয়েছিল? | \|__\| |
| | 0= No treatment<br>1= Oral iron tablets<br>2= Intravenous iron<br>3= Blood transfusion<br>4= Unsure | |
| 17a | Did you take iron folic acid (IFA) tablet in last pregnancy?<br>বিগত গর্ভকালীন সময়ে আপনি আয়রন ফলিক এসিড খেয়েছেন কি? | 0= No (Skip to Q18 a )<br>1= Yes<br>2= Unsure (Skip to Q18 a ) |
| 17 b | How often did you take IFA oral tablet?<br>আপনি কতদিন পরপর আয়রন ফলিক এসিড খেতেন? | \|__\|__\| |
| | 1= Most of days (4-6 days days per week)<br>2= About 3 days per week<br>3= A few days (3-5 days) per month<br>4= Only a few days (3-5 days) during whole pregnancy<br>5= Unsure<br>6= Everyday | |
| 17c | Who prescribed the IFA oral tablet?<br>আপনাকে কে আয়রন ফলিক এসিড খেতে বলেছিল? | \|__\|__\| |
| | 0= By self from a drug store<br>1= Graduate doctor<br>2= Nurse/Midwife/Paramedic<br>3= Family Welfare Visitor (FWV)<br>4= Sub-Assistant Community Medical Officer (SACMO)<br>5= Community Health Care Provider (CHCP)<br>6= Family Welfare Assistant (FWA) | |

| | | |
|---|---|---|
| | 7= Health Assistant (HA)<br>8= NGO worker<br>9= Unqualified doctor<br>10= Drug seller<br>11= Unsure<br>99=Others, Specify_____ | |
| 18a | Did you receive intravenous iron in any previous pregnancy?<br>(Prompt mother for recalling)<br>বিগত গর্ভাবস্থায় আপনি কি শিরায় দেয়া আয়রণ গ্রহন করেছেন? কয় ডোজ? | 0= No (Skip to Q19)<br>1= Yes<br>2= Unsure (Skip to Q19) |
| 18 b | How many infusions (doses) did you receive in a single pregnancy?<br>কয় ডোজ নিয়েছিলেন? | Number: \|___\|___\| |
| 18c | Where did you receive the intravenous iron infusion?<br>আপনি কোথায় শিরায় দেয়া আয়রণ গ্রহন করেছেন? | \|__\|__\| |
| | 0= Drug seller/store<br>1=Private practitioner's Chamber,<br>2= Private facilities (clinics/hospitals)<br>3= Public medical college hospital<br>4= Private medical college hospital<br>5= Tertiary hospital<br>6=District Hospital/sadar hospital<br>7=Upazila Health Complex (UHC)<br>8= Union Sub-center<br>9= Union Health & Family Welfare Center (UH& FWC)<br>10= Satellite clinic<br>11=Community Clinic<br>12= Maternal and Child Welfare Centre<br>13= NGO facilities<br>14= At home<br>99=Others, Specify______ | |
| | **Service use during current pregnancy**<br>**বর্তমানে গর্ভকালীন সেবা গ্রহন** | |
| | | |
| 19 | Have you had any illness so far in this pregnancy? *(Choose all that apply)*<br>এই গর্ভাবস্থায় জানামতে আপনার কোন অসুখ/অসুস্থ্যতা আছে? | 0= No, 1= Yes |
| | *0= Cough* | \|___\| |
| | *1= High Fever* | \|___\| |
| | *2= Dizziness* | \|___\| |
| | *3= Severe headache* | \|___\| |
| | *4= Blurred vision* | \|___\| |
| | *5= Vaginal bleeding* | \|___\| |
| | *6= Convulsion* | \|___\| |
| | *7= Vomiting* | \|___\| |
| | *8= Vaginal discharge* | \|___\| |
| | *9= Diagnosed case of Diabetes* | \|___\| |
| | *10= Diagnosed case of Hypertension* | \|___\| |
| | *99= Other; specify* | \|___\|__\| |
| 20 | Have you ever visited to a provider/health center for your current pregnancy care?<br>আপনি বর্তমান গর্ভকালীন সেবা গ্রহনের জন্য কারো কাছে /স্বাস্থ্য কেন্দ্রে কখনও গেছেন কি? | \|___\| 0= No [skip to Q26], 1= Yes |
| 21 | When have you first visited for ANC in this pregnancy?<br>বর্তমান গর্ভাবস্থায় আপনি কবে প্রথম ANC সাক্ষাতের জন্য গেছেন? | **at** \|__\|__\| week of pregnancy |
| 22 | Where have you gone to receive this service? | \|___\|__\| |

| | | |
|---|---|---|
| | আপনি এই সেবা নিতে কোথায় গেছেন? | |
| | 1=Private practitioner's Chamber,<br>2= Private facilities (clinics/hospitals)<br>3= Public medical college hospital<br>4= Private medical college hospital<br>5= Tertiary hospital<br>6=District Hospital/Sadar hospital<br>7=Upazila Health Complex (UHC)<br>8= Union Sub-center<br>9= Union Health & Family Welfare Center (UH& FWC)<br>10= Satellite clinic<br>11=Community Clinic<br>12= Maternal and Child Welfare Centre<br>13= NGO facilities<br>14= At home<br>99=Other, Specify | |
| 23 | **Why have you selected this health services? Match with listed responses (a to i) and record in likert scale)**<br>কেন আপনি এই স্বাস্থ্য সেবা কেন্দ্র বেছে নিয়েছেন? | **Response**<br>0=Strongly agree, 1=Agree, 2=Not sure, 3=Disagree, 4=Strongly disagree |
| | a. *Health service was easy to reach* \|___\|<br>এই স্বাস্থ্য সেবাকেন্দ্রে সহজে যাওয়া যায় | ০ = পুরাপুরি একমত, ১ = একমত, ২ = নিশ্চিত নয়, ৩ = একমত নই, ৪ = মোটেই একমত নই |
| | b. *I trust the service providers* \|___\|<br>এখানকার সেবা প্রদানকারীদের বিশ্বাস করি | ০ = পুরাপুরি একমত, ১ = একমত, ২ = নিশ্চিত নয়, ৩ = একমত নই, ৪ = মোটেই একমত নই |
| | c. *The health service/provider understands my needs* \|___\|<br>স্বাস্থ্য সেবা প্রদানকারী আমার প্রয়োজন বোঝে | ০ = পুরাপুরি একমত, ১ = একমত, ২ = নিশ্চিত নয়, ৩ = একমত নই, ৪ = মোটেই একমত নই |
| | d. *The service provided is high quality* \|___\|<br>এরা উচ্চমানের সেবা প্রদান করে | ০ = পুরাপুরি একমত, ১ = একমত, ২ = নিশ্চিত নয়, ৩ = একমত নই, ৪ = মোটেই একমত নই |
| | e. *I don't have to wait to get seen* \|___\|<br>এখানে দেখানোর জন্য আমাকে অপেক্ষা/দেরী করতে হয়না | ০ = পুরাপুরি একমত, ১ = একমত, ২ = নিশ্চিত নয়, ৩ = একমত নই, ৪ = মোটেই একমত নই |
| | f. *The service is clean* \|___\|<br>এখানকার সেবা পরিচ্ছন্ন/ ওরা যা বলে তা করে | ০ = পুরাপুরি একমত, ১ = একমত, ২ = নিশ্চিত নয়, ৩ = একমত নই, ৪ = মোটেই একমত নই |
| | g. *My privacy is respected* \|___\|<br>এখানে গোপনীয়তা বজায় রাখে | ০ = পুরাপুরি একমত, ১ = একমত, ২ = নিশ্চিত নয়, ৩ = একমত নই, ৪ = মোটেই একমত নই |
| | h. *Their service has everything needed to provide my care* \|___\|<br>আমার সেবার/যত্নের জন্য প্রয়োজনীয় সব ধরনের ব্যবস্থা এখানে আছে | ০ = পুরাপুরি একমত, ১ = একমত, ২ = নিশ্চিত নয়, ৩ = একমত নই, ৪ = মোটেই একমত নই |
| | i. *The service is cheap or free* \|___\|<br>এদের সেবা/ সার্ভিসের খরচ কম  বা বিনামূল্যে পাওয়া যায় | ০ = পুরাপুরি একমত, ১ = একমত, ২ = নিশ্চিত নয়, ৩ = একমত নই, ৪ = মোটেই একমত নই |
| 24 | Which factor has been most important in your decision to select the services (select letter a-i)<br>উপরে উল্লেখিত কোন কারনটিকে আপনার সিদ্ধান্ত নেওয়ার ক্ষেত্রে সবচেয়ে গুরুত্বপূর্ণ বলে আপনি মনে করেন? | \|__\| |
| 25 | Who is providing you care during your pregnancy?<br>ওখানে কোন স্বাস্থ্য সেবা প্রদানকারী আপনাকে সেবা প্রদান করেছে? | \|__\|__\| |
| | 1= Graduate doctor<br>2= Nurse/Midwife/Paramedic<br>4= Family Welfare Visitor (FWV)<br>5= Sub-Assistant Community Medical Officer (SACMO)<br>6= Community Health Care Provider (CHCP) [a= CSBA, b= Non CSBA]<br>7= Family Welfare Assistant (FWA) [a= CSBA, b= Non CSBA]<br>8= Health Assistant (HA) [a= CSBA, b= Non CSBA]<br>9= NGO worker<br>10= Unqualified doctor<br>11= Unsure<br>99=Other, Specify | |

| | | |
|---|---|---|
| 26 | Are you taking IFA (Iron-Folic acid) tablet in current pregnancy? When have you started ?<br>বর্তমানে গর্ভাবস্থায় আপনি কি IFA (আয়রণ- ফলিক এসিড) গ্রহন করেছেন? কত সপ্তাহে শুরু করেছেন? | \|___\| 0=No, 1=Yes for \|___\|___\| weeks |
| 27 | How often do you take IFA oral tablet?<br>আপনি কতদিন পরপর আয়রন ফলিক এসিড খান? | \|__\| |
| | 1= Most of days (4-6 days days per week)<br>2= About 3 days per week<br>3= A few days (3-5 days) per month<br>4= Only a few days (3-5 days) during whole pregnancy<br>5= Unsure<br>6= Everyday | |
| 28 | Who have prescribed this IFA oral tablet?<br>আপনাকে কে আয়রন ফলিক এসিড খেতে বলেছিল? | \|__\|__\| |
| | 0= By self from a drug store<br>1= Graduate doctor<br>2= Nurse/Midwife/Paramedic<br>3= Family Welfare Visitor (FWV)<br>4= Sub-Assistant Community Medical Officer (SACMO)<br>5= Community Health Care Provider (CHCP)<br>6= Family Welfare Assistant (FWA)<br>7= Health Assistant (HA)<br>8= NGO worker<br>9= Unqualified doctor<br>10= Drug seller<br>11= Unsure<br>99=Other, Specify | |
| 29 | Have you taken any intravenous iron in current pregnancy?<br>বর্তমানে গর্ভাবস্থায় আপনি কি শিরায় দেয়া আয়রণ গ্রহন করেছেন? | \|___\| 0=No [go to Section E], 1=Yes, 2=Not sure [go to Section E ] |
| 30 | How many infusions (doses) have you received in this pregnancy?<br>কয় ডোজ? | Number: \|___\|___\| |

## Section E: Nutritional Knowledge and Practice

*Indicate whether each statement below is True or False. Use 1 for "True", 2 for "False" and 3 for "I don't know."*

| | STATEMENT | RESPONSE |
|---|---|---|
| 1 | It is very important to eat a variety of foods during pregnancy.<br>গর্ভাবস্থায় বিভিন্ন রকমের খাবার খাওয়া খুব জরুরী। | \|___\| |
| 2 | Good nutrition before conception is not necessary.<br>গর্ভাধারনের পূর্বে ভাল পুষ্টি আবশ্যক নয়। | \|___\| |
| 3 | Eating fruits and vegetables during pregnancy increases the chances of being anaemic.<br>গর্ভাবস্থায় ফলমূল ও শাকসব্জি খেলে রক্তাল্পতা হওয়ার ঝুঁকি বাড়ে। | \|___\| |
| 4 | Iron/folic acid supplements given during ANC are detrimental to blood production.<br>গর্ভকালীন সময়ে আয়রণ/ ফলিক এসিড ট্যাবলেট খেলে তা রক্ত তৈরিতে ব্যাঘাত ঘটায়। | \|___\| |
| 5 | Blood loss during pregnancy is normal and hence cannot contribute to anaemia.<br>গর্ভাবস্থায় রক্তক্ষরন স্বাভাবিক, এতে রক্তাল্পতা হয়না। | \|___\| |
| 6 | Treating infections (e.g. diarrhea, UTI) during pregnancy can reduce the chance of being anaemic. | \|___\| |

| | | |
|---|---|---|
| | গর্ভাবস্থায় যেমন- ডায়রিয়া,প্রস্রাবে ইনফেকশনের চিকিৎসা করলে রক্তাল্পতা কম হয়। | |
| 7 | It is advisable for women who are pregnant to stop eating animal source foods (e.g. poultry, fish, meat).<br>গর্ভবতী মহিলাদের প্রানীজ খাবার (যেমন- মুরগী, মাছ, মাংস) না খেতে উপদেশ দেয়া উচিত | \|___\| |
| 8 | Eating more in pregnancy increases the size of foetus which may cause difficulty during delivery<br>গর্ভকালীন সময়ে বেশী বেশী খাবার খেলে গর্ভের শিশুর আকার বড় হয়ে প্রসব/ ডেলিভারীর সময় সমস্যা করতে পারে | \|___\| |

## Section F: Edinburgh Postnatal Depression Scale (EPDS)

(**সেকশন F ঃ** পোষ্টনেটাল ডিপ্রেশন স্কেল)

আমরা জানতে পেরেছি সম্প্রতি আপনি গর্ভবতী হয়েছেন। আপনাকে অভিনন্দন জানাই। আমরা জানতে চাচ্ছি আপনার এখন কেমন লাগছে। শুধু আজকে আপনার কেমন লাগছে তা নয় বরং **গত এক সপ্তাহ** ধরে আপনার কেমন অনুভব হচ্ছে তা কি দয়া করে আমাদেরকে বলবেন। এজন্য আপনাকে ১০টি প্রশ্ন করবো। প্রতিটি প্রশ্নের ৪টি করে উত্তর থাকবে, যে উত্তরটা আপনার সঙ্গে মিলে যাবে বা কাছাকাছি হবে সেটাই বলুন।

একটি উদাহরন দিচ্ছি

*আপনি আনন্দে ছিলেন ঃ*

- ○ হ্যাঁ, সব সময়ই
- ● হ্যাঁ, বেশিরভাগ সময়ই
- ○ না, প্রায়ই না
- ○ না, একেবারেই না

- এটার অর্থ হচ্ছে ‘আপনি গত সপ্তায় বেশিরভাগ সময় আনন্দে ছিলেন’ এভাবে পরবর্তি প্রশ্নগুলোর উত্তর দিন।

| | | |
|---|---|---|
| প্রশ্ন ঃ ১ | | **Score** |
| | আপনি হাসতে পেরেছেন এবং হাসি - তামাসা উপভোগ করতে পেরেছেন<br><br>I have been able to laugh and see the funny side of things | **\|___\|** |
| | 0= হ্যাঁ, আগের মতই<br><br>As much as I always could | |
| | 1= হ্যাঁ, আগের মত ততটা না<br><br>Not quite so much now | |
| | 2= না, এখন ততটা না<br><br>Definitely not so much now | |
| | 3= না, একেবারেই না<br><br>Not at all | |
| | | |
| প্রশ্ন ঃ ২ | আপনি সবকিছু থেকে আনন্দ পাওয়ার আশায় থেকেছেন | |

| | | |
|---|---|---|
| | I have looked forward with enjoyment to things | \|___\| |
| | 0= হ্যাঁ, আগের মতই<br><br>As much as I ever did | |
| | 1= হ্যাঁ, আগের চেয়ে কিছু কম<br><br>Rather less than I used to | |
| | 2= না, আগের চেয়ে অনেক কম<br><br>Definitely less than I used to | |
| | 3= না, একেবারেই না<br><br>Hardly at all | |
| | | |
| প্রশ্ন ঃ ৩ | কোন কিছু ঠিকমত না হলে আপনি নিজেকে অ-যথাই দোষ দিয়ে থাকেন<br><br>I have blamed myself unnecessarily when things went wrong | \|___\| |
| | 3= হ্যাঁ, বেশিরভাগ সময়<br><br>Yes, most of the time | |
| | 2= হ্যাঁ, মাঝে মাঝে<br><br>Yes, some of the time | |
| | 1= না, খুব বেশি না<br><br>Not very often | |
| | 0= না, কখনোই না<br><br>No, never | |
| | | |
| প্রশ্ন ঃ ৪ | আপনি এমনিতেই দুশ্চিন্তা করে থাকেন বা ঘাবড়িয়ে যান<br><br>I have been anxious or worried for no good reason | \|___\| |
| | 0= না, কখনোই না<br><br>No, not at all | |
| | 1= না, খুবই কম<br><br>Hardly ever | |

| | | |
|---|---|---|
| | 2= হ্যাঁ, মাঝে মাঝে<br><br>Yes, sometimes | |
| | 3= হ্যাঁ, প্রায়ই<br><br>Yes, very often | |
| | | |
| প্রশ্ন ঃ ৫ | আপনি এমনিতেই ভয় পেয়েছেন বা আতঙ্কিত হয়েছেন<br><br>I have felt scared or panicky for no very good reason | \|___\| |
| | 3= হ্যাঁ, খুব বেশি<br><br>Yes, quite a lot | |
| | 2= হ্যাঁ, মাঝে মাঝে<br><br>Yes, sometimes | |
| | 1= না, বেশি না<br><br>No, not much | |
| | 0= না, একেবারেই না<br><br>No, not at all | |
| | | |
| প্রশ্ন ঃ ৬ | সবকিছু আপনার কাছে বোঝা মনে হয়েছে এবং আপনি তা মানিয়ে নিতে পারছেন না<br><br>Things have been getting on top of me | \|___\| |
| | 3= হ্যাঁ, বেশিরভাগ সময়ে মানিয়ে নিতে পারছেন না<br><br>Yes, most of the time I haven’t been able to cope at all | |
| | 2= হ্যাঁ, মাঝে মাঝে মানিয়ে নিতে পারছেন না<br><br>Yes, sometimes I haven’t been coping as well as usual | |
| | 1= না, বেশির ভাগ পারছেন<br><br>No, most of the time I have coped quiet well | |
| | 0= না, আপনি সব সময় ভালভাবে মানিয়ে নিতে পারছেন<br><br>No, I have been coping as well as ever | |
| | | |

| | | |
|---|---|---|
| প্রশ্ন ঃ ৭ | আপনার মনটা এতোই খারাপ ছিল যে, আপনার ঘুমের অসুবিধা হয়েছে<br><br>I have been so unhappy that I have had difficulty sleeping | I___I |
| | 3= হ্যাঁ, বেশিরভাগ সময়ই<br><br>Yes, most of the time | |
| | 2= হ্যাঁ, মাঝে মাঝে<br><br>Yes, sometimes | |
| | 1= না, প্রায়ই না<br><br>Not very often | |
| | 0= না, একেবারেই না<br><br>No, not at all | |
| | | |
| প্রশ্ন ঃ ৮ | আপনার নিজেকে দুঃখী বা অসহায় মনে হয়েছে<br><br>I have felt sad or miserable | I___I |
| | 3= হ্যাঁ, বেশিরভাগ সময়ই<br><br>Yes, most of the time | |
| | 2= হ্যাঁ, প্রায় প্রায়ই<br><br>Yes, quite often | |
| | 1= না, প্রায়ই না<br><br>Not very often | |
| | 0= না, একেবারেই না<br><br>No, not at all | |
| | | |
| প্রশ্ন ঃ ৯ | আপনার মনটা এতোই খারাপ ছিল যে আপনি কেঁদেছেন<br><br>I have been so unhappy that I have been crying | I___I |
| | 3= হ্যাঁ, বেশিরভাগ সময়ই<br><br>Yes, most of the time | |
| | 2= হ্যাঁ, প্রায় প্রায়ই | |

| | | |
|---|---|---|
| | Yes, quite often | |
| | 1= হ্যাঁ, কখনো কখনো<br><br>Only occasionally | |
| | 0= না, কখনোই না<br><br>No, never | |
| | | |
| প্রশ্ন ঃ ১০ | আপনি নিজেই নিজের ক্ষতি করার কথা ভেবেছেন<br><br>The thought of harming myself has occurred to me | \|___\| |
| | 3= হ্যাঁ, প্রায়ই<br><br>Yes, quite often | |
| | 2= হ্যাঁ, মাঝে মাঝে<br><br>Sometimes | |
| | 1= না, খুবই কম<br><br>Hardly ever | |
| | 0= কখনোই না<br><br>Never | |
| | | |

## Section G: Woman's autonomy

**(সেকশন G ঃ নারীর সিদ্ধান্ত নেয়ার অধিকার)**

১.আপনার উপার্জিত অর্থ কিভাবে ব্যয় হবে তা সাধারনত কে ঠিক করেন? /____/

**Who usually decides how the money you earn will be used?**

১ = আপনি, ২ = স্বামী, ৩ = আপনি এবং আপনার স্বামী মিলিত ভাবে, ৪ = অন্য কেউ, ৫ =মায়ের নিজের কোন রোজকার/আয় নেই

Respondent=1, Husband=2, Respondent and Husband jointly=3, Someone else=4, Mother doesn't have own income=5

২. আপনার স্বাস্থ্য সেবার ক্ষেত্রে কে সাধারনত সিদ্ধান্ত নেন? /____/

**Who usually makes decisions about health care for yourself:**

১ = আপনি, ২ = স্বামী, ৩ = আপনি এবং আপনার স্বামী মিলিত ভাবে, ৪ = অন্য কেউ, ৯৯ = অন্যান্য, নির্দিষ্ট করুন ...................

Respondent=1, Husband=2, Respondent and Husband jointly=3, Someone else=4, Other=99, Specify..

৩. পরিবারের বড় ধরনের কেনাকাটার বিষয়ে কে সাধারনত সিদ্ধান্ত নেন? /___/

**Who usually makes decisions about making major household purchase:**

১ = আপনি, ২ = স্বামী, ৩ = আপনি এবং আপনার স্বামী মিলিত ভাবে, ৪ = অন্য কেউ, ৯৯ = অন্যান্য, নির্দিষ্ট করুন ...............................

Respondent=1, Husband=2, Respondent and Husband jointly=3, Someone else=4, Other=99, Specify..

৪. আপনার পরিবার বা আত্নীয় স্বজনের সাথে সাক্ষাতের বিষয়ে কে সাধারনত সিদ্ধান্ত নেন? /___/

**Who usually makes decisions about visit to your family or relatives:**

১ = আপনি, ২ = স্বামী, ৩ = আপনি এবং আপনার স্বামী মিলিত ভাবে, ৪ = অন্য কেউ, ৯৯ = অন্যান্য, নির্দিষ্ট করুন Respondent=1, Husband=2, Respondent and Husband jointly=3, Someone else=4, Other=99, Specify..

৫. আপনার সন্তানের স্বাস্থ্য সেবার ক্ষেত্রে সাধারনত কে সিদ্ধান্ত নেন? /___/

**Who usually makes decisions about your child's health care:**

১ = আপনি, ২ = স্বামী, ৩ = আপনি এবং আপনার স্বামী মিলিত ভাবে, ৪ = অন্য কেউ, ৯৯ = অন্যান্য, নির্দিষ্ট করুন Respondent=1, Husband=2, Respondent and Husband jointly=3, Someone else=4, Other=99, Specify..

৬. স্বাস্থ্য কেন্দ্র বা হাসপাতালে আপনি কি একাই যান, নাকি সাথে কোন বাচ্চা নিয়ে যেতে হয়? /___/

**Do you go to a health center or hospital alone or with your young children?**

১ = হ্যাঁ, একাই; ২ = হ্যাঁ, ছোট বাচ্চা সাথে থাকে, ৩= না, ৯৯ = অন্যান

্য, নির্দিষ্ট করুন ................................

Yes, Alone=1, Yes, with children=2, No=3, Other=99, Specify……..

| | **Section H: Birth Preparedness and Complication Readiness** | |
|---|---|---|
| 1 | Have you planned or made decision on place of delivery?<br>আপনি কি বাচ্চা প্রসবের (ডেলিভারির) জন্য কোন স্থান ঠিক করেছেন বা সি্দ্ধান্ত নিয়েছেন? | \|___\| 1= Yes, 0= No |
| 2 | Do you have any arrangement for skilled attendance at delivery?<br>বাচ্চা প্রসবের (ডেলিভারির) জন্য আপনার কি দক্ষ ধাত্রী ঠিক করা আছে? | \|___\| 1= Yes, 0= No |
| 3 | Do you have savings for the delivery or if needed in case of emergency?<br>বাচ্চা প্রসব (ডেলিভারির) জন্য বা জরুরী ভিত্তিতে প্রয়োজন হলে আপনার কি অর্থ সঞ্চিত আছে? | \|___\| 1= Yes, 0= No |
| 4 | Have you identified/ arranged transportation in case of emergency?<br>জরুরী প্রয়োজন পড়লে আপনার গাড়ী/বাহন কি ঠিক করা আছে? | \|___\| 1= Yes, 0= No |
| 5 | Do you have any arrangement for a potential blood donor?<br>আপনার কি একজন সম্ভাব্য রক্তদাতা ঠিক করা আছে? | \|___\| 1= Yes, 0= No |

| | | |
|---|---|---|
| 6 | Can you tell me the signs when to go to nearby hospital? (don't promote, just ask " say few more"<br>আপনি জানেন কি কোন লক্ষনগুলি দেখা দিলে নিকটবর্তী হাসপাতালে যেতে হবে? | \|___\| 1= Yes, 0= No |
| | *1) Vaginal bleeding* | \|___\| |
| | *2) Severe headache/ blurring of vision* | \|___\| |
| | *3) Convulsion* | \|___\| |
| | *4) Delayed labour* | \|___\| |
| | *5) High fever* | \|___\| |

## Section I : WASH questionnaire

**(সেকশন I ঃ WASH তথ্য)**

| | প্রশ্ন | | রেসপন্স | কোড | নির্দেশনা |
|---|---|---|---|---|---|
| ১ | আপনার পরিবারের সদস্যদের খাবার পানির প্রধান উৎস কি?<br>What is the main source of drinking-water for members of your household? | 1 | ঘরের ভিতরে পাইপ লাইনের পানি<br>Piped water into dwelling | \|____\| | প্রশ্ন ৪ এ যান |
| | | 2 | উঠানে/ প্লটের পাইপের পানি<br>Piped water to yard/plot | \|____\| | প্রশ্ন ৪ এ যান |
| | | 3 | সরকারী কল/ স্ট্যান্ড পাইপ<br>Public tap/standpipe | \|____\| | প্রশ্ন ২ এ যান |
| | | 4 | টিউবওয়েল/ নলকূপ<br>Tubewell/borehole | \|____\| | প্রশ্ন ২ এ যান |
| | | 5 | ধরে রাখা বৃষ্টির পানি<br>Rainwater collection | \|____\| | প্রশ্ন ২ এ যান |
| | | 6 | বোতলজাত পানি<br>Bottled water | \|____\| | প্রশ্ন ১(ক) এ যান Go to Q1A |
| | | 7 | ভূমির উপরিতলের পানি (নদী, বাঁধ,পুকুর, ঝর্ণা, সেচের)<br>Surface water (river, dam, lake, pond, stream, canal, irrigation channels) | \|____\| | প্রশ্ন ২ এ যান |
| | | 99 | অন্যান্য (উল্লেখ করুন--- <br>Other (specify) | \|____\| | প্রশ্ন ২ এ যান |
| | | | | | |
| ১.ক | আপনার পরিবারের সদস্যদের অন্যান্য কাজের জন্য যেমনঃ রান্না ও হাত ধোয়ার জন্য ব্যবহৃত পানির প্রধান উৎস কি?<br>What is the main source of water used by your household for other purposes, such as cooking and hand washing? | 1 | ঘরের ভিতরে পাইপ লাইনের পানি<br>Piped water into dwelling | \|____\| | প্রশ্ন ৪ এ যান |
| | | 2 | উঠানে/ প্লটের পাইপের পানি<br>Piped water to yard/plot | \|____\| | প্রশ্ন ৪ এ যান |
| | | 3 | সরকারী কল/ স্ট্যান্ড পাইপ<br>Public tap/standpipe | \|____\| | প্রশ্ন ২ এ যান |
| | | 4 | টিউবওয়েল/ নলকূপ<br>Tubewell/borehole | \|____\| | প্রশ্ন ২ এ যান |
| | | 5 | ধরে রাখা বৃষ্টির পানি<br>Rainwater collection | \|____\| | প্রশ্ন ২ এ যান |
| | | 6 | ভূমির উপরিতলের পানি(নদী,বাঁধ,পুকুর, ঝর্ণা, সেচের) | \|____\| | প্রশ্ন ২ এ যান |

| | | | | | |
|---|---|---|---|---|---|
| | | | Surface water (river, dam, lake, pond, stream, canal, irrigation channels) | | |
| | | 99 | অন্যান্য (উল্লেখ করুন)---- Other (specify) | \|____\| | প্রশ্ন ২ এ যান |
| | | | | | |
| ২ | পানির জন্য যাওয়া ও ফিরে আসতে কত সময় লাগে? How long does it take to go there, get water, and come back? | 1 | উল্লেখ করুন কত মিনিট: (And specify number of minutes ______) ।____\|____\| | | প্রশ্ন ৩ এ যান |
| | | 2 | বাড়ির সীমানায়/ বাড়ির ভিতরেই উৎস Water on premises | \|____\| | প্রশ্ন ৪ এ যান |
| | | 3 | জানিনা Don't know | \|____\| | প্রশ্ন ৩ এ যান |
| | | | | | |
| ৩ | সাধারনতঃ কে ঐ উৎস থেকে আপনার বাড়ির জন্য পানি নিয়ে আসে? ( নির্দিষ্ট ব্যক্তির পাশের কোড বৃত্তাকার করুন) Who usually goes to this source to fetch the water for your household? | 1 | প্রাপ্ত বয়স্কা মহিলা Adult woman | \|____\| | প্রশ্ন ৪ এ যান |
| | | 2 | প্রাপ্ত বয়স্ক পুরুষ Adult man | \|____\| | প্রশ্ন ৪ এ যান |
| | | 3 | কন্যা শিশু(১৫-১৭ বছরের) Female child (15-17 years) | \|____\| | প্রশ্ন ৪ এ যান |
| | | 4 | ছেলে শিশু(১৫-১৭ বছরের) Male child (15-17 years) | \|____\| | প্রশ্ন ৪ এ যান |
| | | 5 | কন্যা শিশু(১৫ বছরের নীচে) Female child (<15 years) | \|____\| | প্রশ্ন ৪ এ যান |
| | | 6 | ছেলে শিশু(১৫ বছরের নীচে) Male child (<15 years) | \|____\| | প্রশ্ন ৪ এ যান |
| | | 7 | জানিনা Don't know | \|____\| | প্রশ্ন ৪ এ যান |
| ৪ | পানিকে পানের উপযোগী করার জন্য আপনি কি কোন ভাবে পরিশোধন করেন Do you treat your water in any way to make it safer to drink? | 1 | হ্যাঁ Yes | \|____\| | প্রশ্ন ৫ এ যান |
| | | 0 | না No | \|____\| | প্রশ্ন ৬ এ যান |
| | | 2 | জানিনা Don't know | \|____\| | প্রশ্ন ৬এ যান |
| | | | | | |
| ৫ | সাধারনতঃ আপনি নিরাপদ/ বিশুদ্ধ পানি পান করার জন্য কি করেন? এছাড়াও অন্য কিছু করেনকি? একাধিক উত্তর গ্রহনযোগ্য What do you usually do to the water to make it safer to drink? Anything else? Record all items mentioned | 1 | ফুটাই Boil | \|____\| | প্রশ্ন ৬ এ যান |
| | | 2 | ব্লিচিং/ ক্লোরিন যোগ করি Add bleach/chlorine | \|____\| | প্রশ্ন ৬এ যান |
| | | 3 | কাপড় দিয়ে ছেঁকে নেই Strain it through a cloth | \|____\| | প্রশ্ন ৬ এ যান |
| | | 4 | পানির ফিল্টার ব্যবহার করি (সিরামিক,বালু, একাধিক উপাদানের মিশ্রণ,ইত্যাদি) Use a water filter (ceramic, sand, composite, etc.) | \|____\| | প্রশ্ন ৬ এ যান |
| | | 5 | সৌর পদ্ধতিতে জীবানুমুক্ত করি Solar disinfection | \|____\| | প্রশ্ন ৬ এ যান |
| | | 6 | রেখে দিয়ে তলানী ফেলি Let it stand and settle | \|____\| | প্রশ্ন ৬ এ যান |
| | | 7 | জানিনা | \|____\| | প্রশ্ন ৬ এ যান |

<table>
<tr><td></td><td></td><td></td><td>Don’t know</td><td></td><td></td></tr>
<tr><td></td><td></td><td>99</td><td>অন্যান্য(উল্লেখ করুন)-----<br>Other (specify)</td><td>|____|</td><td>প্রশ্ন ৬এ যান</td></tr>
<tr><td rowspan="15">৬</td><td rowspan="15">আপনার পরিবারের সদস্যরা সাধারনত কি ধরনের পায়খানা ব্যবহার করে? (ট্যাংকির পানি ফ্লাশ করে বা পানি সজোরে ঢেলে দিলে এটা কোথায় যায়?)<br>What kind of toilet facility do members of your household usually use? If “flush” or “pour flush” probe: Where does it flush to?</td><td>1</td><td>পাইপ পয়ঃনিষ্কাশন পদ্ধতিতে<br>Flush to piped sewer system</td><td>|____|</td><td>প্রশ্ন ৭ এ যান</td></tr>
<tr><td>2</td><td>সেপ্টিক ট্যাঙ্কি<br>Flush to septic tank</td><td>|____|</td><td>প্রশ্ন ৭ এ যান</td></tr>
<tr><td>3</td><td>টয়লেটের গর্তে<br>Flush to pit latrine</td><td>|____|</td><td>প্রশ্ন ৭ এ যান</td></tr>
<tr><td>4</td><td>অন্য কোথাও<br>Flush to somewhere else</td><td>|____|</td><td>প্রশ্ন ৭ এ যান</td></tr>
<tr><td>5</td><td>কোথায় যায় জানিনা<br>Flush, Don’t know where</td><td>|____|</td><td>প্রশ্ন ৭ এ যান</td></tr>
<tr><td>6</td><td>বাতাস চলাচলের সুবিধা আছে এমন পিট/গর্তে (স্লাব সহ)<br>Ventilated improved pit latrine</td><td>|____|</td><td>প্রশ্ন ৭এ যান</td></tr>
<tr><td>7</td><td>পিট/গর্তযুক্ত পায়খানায় (স্লাব সহ)<br>Pit latrine with slab</td><td>|____|</td><td>প্রশ্ন ৭এ যান</td></tr>
<tr><td>8</td><td>স্লাব বা ঢাকনাবিহীন পিট/গর্তযুক্ত পায়খানায়<br>Pit latrine without slab/open pit</td><td></td><td></td></tr>
<tr><td>9</td><td>কম্পোষ্ট বা সার টয়লেট<br>Composting toilet</td><td>|____|</td><td>প্রশ্ন ৭ এ যান</td></tr>
<tr><td>10</td><td>বালতি<br>Bucket toilet</td><td>|____|</td><td>প্রশ্ন ৭ এ যান</td></tr>
<tr><td>11</td><td>ঝুলন্ত পায়খানা<br>Hanging toilet/Hanging latrine</td><td>|____|</td><td>প্রশ্ন ৭ এ যান</td></tr>
<tr><td>12</td><td>কোন সুবিধা নাই/ ঝোঁপ ঝার বা মাঠে<br>No facility/Bush/Field</td><td>|____|</td><td>প্রশ্ন ৯ এ যান Go to Q9</td></tr>
<tr><td>99</td><td>অন্যান্য------<br>Other, Specify</td><td>|____|</td><td>প্রশ্ন ৭ এ যান</td></tr>
<tr><td></td><td></td><td></td><td></td></tr>
<tr><td rowspan="3">৭</td><td rowspan="3">আপনার এই টয়লেট কি অন্য পরিবারের সদস্যরাও ব্যবহার করেন?<br>Do you share this facility with other households?</td><td>1</td><td>হ্যাঁ<br>Yes</td><td>|____|</td><td>প্রশ্ন ৮ এ যান</td></tr>
<tr><td>0</td><td>না<br>No</td><td>|____|</td><td>প্রশ্ন ৯ এ যান</td></tr>
<tr><td></td><td></td><td></td><td></td></tr>
<tr><td rowspan="3">৮</td><td rowspan="3">অন্য কয়টি পরিবার এই টয়লেট ব্যবহার করেন?<br>(পরিবারের সংখ্যা নির্দিষ্ট করুন<br>(১০ পরিবারের বেশী হলে > ১০ লিপিবদ্ধ করুন):<br>How many other households use this toilet facility?</td><td>1</td><td>|____|____| পরিবার<br>|____|____| family</td><td>|____|</td><td>প্রশ্ন ৯ এ যান</td></tr>
<tr><td>2</td><td>>১০ পরিবার<br>>10 family</td><td>|____|</td><td>প্রশ্ন ৯ এ যান</td></tr>
<tr><td>3</td><td>জানিনা<br>Don’t know</td><td>|____|</td><td></td></tr>
<tr><td>৯</td><td></td><td>1</td><td>শিশু টয়লেট/ ল্যাট্রিন ব্যবহার করেছিল<br>Child used toilet/latrine</td><td>|____|</td><td>প্রশ্ন ১০ এ যান</td></tr>
</table>

| | | | | | |
|---|---|---|---|---|---|
| | (আপনার শিশু) যখন শেষ বার পায়খানা করেছিল ,তখন তা কিভাবে ফেলেছিলেন?<br><br>While a child passed stools, what was done to dispose of the stools? | 2 | টয়লেট/ ল্যাট্রিনে ফেলে দিয়েছি, অল্প পানি দিয়ে ধুয়ে ফেলেছি<br>Put/rinsed into toilet or latrine | \|____\| | প্রশ্ন ১০ এ যান |
| | | 3 | ড্রেইন বা নর্দমার গর্তে ফেলে দিয়েছি<br>Put/rinsed into drain or ditch | \|____\| | প্রশ্ন ১০ এ যান |
| | | 4 | আবর্জনার স্তুপে ফেলে দিয়েছি<br>Thrown into garbage | \|____\| | প্রশ্ন ১০এ যান |
| | | 5 | মাটি চাপা দিয়েছি<br>Buried | \|____\| | প্রশ্ন ১০ এ যান |
| | | 6 | খোলা স্থানে ফেলেছি<br>Left in the open | \|____\| | প্রশ্ন ১০ এ যান |
| | | 7 | জানিনা<br>Don't know | \|____\| | প্রশ্ন ১০ এ যান |
| | | 99 | অন্যান্য, উল্লেখ করুন--------<br>Other (specify) | \|____\| | প্রশ্ন ১০ এ যান |
| | | | | | |
| ১০ | আপনি দয়া করে আমাকে বলুন কোন কোন ক্ষেত্রে হাত ধোয়া আপনার জন্য গুরুত্বপূর্ণ বলে আপনি মনে করেন? (একাধিক উওর হতে পারে, প্রযোজ্য ক্ষেত্রে টিক দিন ।নির্দিষ্ট কিছু বলার জন্য উৎসাহ দিবেন না, বরং মা থেমে গেলে আরও কিছু বলার আছে কিনা জিজ্ঞাসা করতে পারেন। )<br><br>Please tell me all of the occasions when it is important for you to wash your hands (tick responses – do not prompt, but can ask 'Anything else?'): | 1 | খাবার আগে \|_____\|<br>Before eating | \|____\| | |
| | | 2 | বাচ্চাকে খাওয়ানোর আগে \|_____\|<br>Before feeding a child | \|____\| | |
| | | 3 | রান্না করার আগে/ খাবার তৈরী করার আগে \|_____\|<br>Before cooking/preparing food | \|____\| | |
| | | 4 | প্রশ্রাব বা পায়খানার পর বা যে কোন ময়লা ধরলেই<br>After urination/defaecation or after handlining any garbage | \|____\| | |
| | | | | | |
| | | 5 | শিশুর পায়খানা / প্রশ্রাব পরিস্কার বা শিশুর নেপি বা ডায়াপার পরিবর্তন করার পর \|_____\|<br>After cleaning a child who has urinated/defaecated or changing/washing nappy | \|____\| | প্রশ্ন ১১ এ যান |
| | | 6 | বাহিরে থেকে বাড়িতে ফিরে আসার পরই<br>After back to home from outdoors | \|____\| | প্রশ্ন ১১ এ যান |
| ১০ক | (সকল সঠিক উত্তর যোগ করুন) --- সংখ্যাটি ০-৬ পর্যন্ত হবে।<br>TOTAL (add all correct responses) – value should be 0-6 | | | \|____\| | |
| ১১ | আপনি হাত ধোয়ার জন্য সচরাচর কি ব্যবহার করেন?<br>What do you usually use to wash your hands? | 1 | সাবান ও পানি<br>Soap and water | \|____\| | প্রশ্ন ১২ এ যান |
| | | 2 | মাটি/ কাঁদা<br>Mud/clay | \|____\| | প্রশ্ন ১২ এ যান |
| | | 3 | ছাই<br>Ash | \|____\| | প্রশ্ন ১২এ যান |
| | | 4 | শুধু পানি<br>Water only | \|____\| | প্রশ্ন ১২ এ যান |

| | | | | | |
|---|---|---|---|---|---|
| *পরবর্তী প্রশ্ন সমূহ পর্যবেক্ষনের সাথে সম্পর্কিত এবং প্রশ্ন পত্র অংশগ্রহনকারীর বাড়ীতে সম্পন্য করলেই শুধুমাত্র কোড করবেন। (মা কে প্রশ্ন জিজ্ঞাসা করা যাবে না)* | | | | | |
| ১২ | বাড়িতে বা বাড়ির উঠানে কি মানুষের মল আছে?<br>Are there human faeces around the house or in the compound? | 1 | হ্যাঁ Yes | \|____\| | প্রশ্ন ১৩ এ যান |
| | | 0 | না No | \|____\| | প্রশ্ন ১৩ এ যান |
| | | | | | |
| ১৩ | বাড়িতে বা বাড়ির উঠানে কি পশুপাখির গোবর বা বিষ্ঠা আছে?<br>Are there animal faeces around the house or in the compound? | 1 | হ্যাঁ Yes | \|____\| | প্রশ্ন ১৪ এ যান |
| | | 0 | না No | \|____\| | প্রশ্ন ১৪ এ যান |
| | | | | | |
| ১৪ | বাড়িতে বা বাড়ির উঠানে আবর্জনা ফেলার স্তুপ(খোলা বা মাটির গর্তে ) আছে?<br>Is there garbage (open garbage/garbage on the ground) around the house or in the compound? | 1 | হ্যাঁ Yes | \|____\| | |
| | | 0 | না No | \|____\| | জরিপের সমাপ্তি |

| | | |
|---|---|---|
| **Section J: COVID-19 related information** | | |
| 1 | Have you ever been tested and diagnosed as a case of COVID-19 by laboratory test?<br>আপনি কি কখনও কোভিড-১৯ এ আক্রান্ত হয়েছেন, যা পরিক্ষার মাধ্যমে ধরা পড়েছে? | \|__\| 0=Yes tested, but not diagnosed as case [Skip to Q9],<br>1=Yes tested and diagnosed as case,<br>2=Not tested [Skip to Q9] |
| 2 | When you have tested for COVID-19?<br>আপনি কবে কোভিড টেষ্টটি করেছেন? | / /20\|__\| (dd /mm /yyyy) |
| 3 | Were you pregnant at the time of COVID-19 infection?<br>কোভিড-১৯ সংক্রমনের সময় আপনি কি গর্ভবতী ছিলেন? | \|__\| 0=No; If No go to Q5, 1=Yes |
| 4 | What was the gestational week at that time?<br>তখন আপনার গর্ভকালীন সপ্তাহ কত ছিল? | \|___\|___\| weeks |
| 5 | Do you have the following sign-symptoms? Check all that apply (√)<br>আপনার নিম্নের কোন লক্ষনগুলো ছিল? | **Responses** |
| | | \|__\|Cough |
| | | \|__\|Sore throat |
| | | \|__\|Runny nose |
| | | \|__\|Wheezing |
| | | \|__\|Chest pain |
| | | \|__\|Muscle aches (myalgia) |
| | | \|__\|Joint pain (arthralgia) |
| | | \|__\|Fatigue/Malaise |
| | | \|__\|Shortness of breath |
| | | \|__\|Inability to talk |
| | | \|__\|Lower chest wall indrawing |

| | | |
|---|---|---|
| | | \|__\|Pneumonia/ ARDS (Acute respiratory distress syndrome) |
| | | \|__\|Headache |
| | | \|__\|Altered consciousness/confusion |
| | | \|__\|Seizures |
| | | \|__\|Abdominal pain |
| | | \|__\|Nausea |
| | | \|__\|Vomiting |
| | | \|__\|Diarrhea |
| | | \|__\|Conjunctivitis |
| | | \|__\|Skin rash |
| | | \|__\|Skin ulcers |
| | | \|__\|Lymphadenopathy |
| | | \|__\|Loss of sense of smell |
| | | \|__\|Loss of taste |
| | | \|__\|SARDS (Sudden acquired retinal degeneration syndrome) |
| | | \|__\|Others, specify: |
| | | \|__\|No symptom |
| 7 | Did you require hospitalization due to COVID-19 infection?<br>কোভিড-১৯ সংক্রমনের জন্য আপনি কি হাসপাতালে ভর্তি হয়েছিলেন? | \|___\| 0= No, 1= Yes |
| 8 | What is your health status at present?<br>বর্তমানে আপনার শাররীক অবস্থা কেমন? | \|__\| |
| | 1=Recovered | |
| | 0= Not Recovered | |
| 9 | Have you received COVID-19 vaccine?<br>আপনি কি করোনার টিকা নিয়েছেন? | 0=No [Go to Q11] 1=Yes (if yes go to Q-10) |
| 10 | Number of dose:<br>মোট কত ডোজ নিয়েছেন? | \|__\| |
| 10 a | Date of last dose received: ____________<br>শেষ টিকার তারিখ? | |
| 11 | Would you get the COVID-19 vaccine if available?<br>কোভিড বা করোনার ভেক্সিন সহজলভ্য হলে আপনি নিবেন কি? | 0=No [Go to Q12] 1=Yes (Stop here) 2=Unsure [Go to Q12] |
| 12 | What is the reason/reasons Check all that apply (√) | **Responses** |
| | অনুগ্রহকরে না নেওয়ার কারণগুলো জানাবেন কি? [একাধিক উত্তর গ্রহনযোগ্য] | \|__\|Do not think it is needed |
| | | \|__\|Heard or read negative media |
| | | \|__\|Do not know where to get the vaccine |
| | | \|__\|Had a bad experience or reaction with previous vaccintation |
| | | \|__\|Do not know where to get reliable information |
| | | \|__\|Had a bad experience with previous health clinic/vaccinator |
| | | \|__\|Not possible to leave other work (at home or other) |
| | | \|__\|Someone else told me they had a bad reaction |
| | | \|__\|Do not think the vaccine is effective |
| | | \|__\|Someone told me the vaccine was not safe |
| | | \|__\|Concerned abou the side effects |

| | | |
|---|---|---|
| | | \|__\|Fear of needles |
| | | \|__\|Religious reasons |
| | | \|__\|Other (explain) |
| | | |

---

| | **Section K: Drinking water iron measurement** | |
|---|---|---|
| 1 | Date and Time of measurement (hh:mm)**:(in 24hrs format)** | Date: ----/----/---- Time: \|__\|__\|: \|__\|__\| |
| 2 | Water iron level (measured by HACH kit) | \|____\|.\|___\| mg/L |

## To be completed in recruitment site

## Section L: Venous blood haemoglobin measurement in field

*(R=Rupganj, S= Sonargaon, B=Bandar)* **PID:** |__1__|____|____|____|____|

*Group: |___|, 2= Second Trimester, 3= Third Trimester*

*Mothers Name:*________________________________ *Village:*________________________________

1. Date and Time of collection: Date: |___|___|___|___|. |___|___|___|___| Time:|___|___|___|___|
2. Has sample been collected? 1=Yes, required amount collected,2= Insufficient amount 3= Failed to collect, 4= Others—specify____________ |___|
3. Venous blood Hb: |____|____|.|____| g/dl

   3.a. Capillary Hb: |____|____|.|____| g/dl
4. Whole Blood (EDTA) sample collected |___| 1=Yes, 0= No
5. Blood for Serum collected |___| 1=Yes, 0= No

Note:
______________________________________________________________________________

---

Collected by: ________________________________________________________________

---

## To be completed in Field Lab

## Section M: Sample Processing and storage in Lab

1. **PID:** |_1_|___|___|___|___|

2. Date and Time of processing:|___|___|___|___|.|___|___|___|___|Time|___|___|: |___|___|

3. Whole Blood(EDTA) volume : |___|.|___| ml

4. Blood for serum volume : |___|.|___| ml

5. Aliquot no:

6. |_1_|___|___|___|___|**W1** storing box no |___|___|___|, location no |___|___|,|___|.|___|ml

7. |_1_|___|___|___|___|**S1** storing box no |___|___|___|, location no |___|___|,|___|.|___|ml

8. |_1_|___|___|___|___|**S2** storing box no |___|___|___|, location no |___|___|,|___|.|___|ml

9. |_1_|___|___|___|___|**S3** storing box no |___|___|___|, location no |___|___|,|___|.|___|ml

Note: ______________________________________________________________

Processed by: ___________________________________ Signature: _____________

**Please transfer the information to TAB version of the questionnaire and upload to server**

## Section N: Interview outcome

1. Result of the interview: 1=Enrolled, PID|__________ (by scanning),2= Not enrolled

2. Reasons for non enrollment: Absent during enrollment|__| Blood refusal|___] Family disapproval|___| Not interested |___|, Acute illness |____|Others, specify|_______|

## Section O: Supportive document scanning

1. Has the participant shown any health/ pregnancy related papers to scan?
   0=No, 1= Yes

2. Upload |___________|
3. Write special note for this participant if any__________________
