# Supplementary material for: Testing and treating anaemia in pregnant women in Bangladesh: a cross-sectional survey
Source: BMJ Public Health. 2025 Jul 16;3(2):e002167. doi: 10.1136/bmjph-2024-002167 (PMC12273131; doi:10.1136/bmjph-2024-002167)
Supplement: online supplemental file 3 [file bmjph-3-2-s003.docx]

**Online supplemental appendix C – Author reflexivity statement**

1. **How does the study address local research and policy priorities?**

The overall programme: ‘**E**fficacy and **D**emonstration of **I**ntra**V**enous iron for **A**naemia in pregnancy’ (EDIVA) was designed as a research collaboration between research institutes in Bangladesh (icddr,b) and Australia (WEHI, University of Melbourne) to address the persistent high prevalence of anaemia in pregnant women in Bangladesh. The EDIVA programme is supported by the Bangladesh Ministry of Health and Family Welfare, demonstrating its’ alignment with local research and policy priorities. For this particular paper and analysis, the research questions and analysis plan were developed by EV with the guidance of PhD supervisors (KHP, MAB, CV) to fulfil the requirements of her doctoral research and were approved by the local team of researchers in Bangladesh (MIH, SA, QR, MB, MT, BS, JH).

1. **How were local researchers involved in study design?**

The survey analysed in this study was developed by icddr,b researchers with support from colleagues at WEHI and the University of Melbourne. Questions included in the survey were therefore culturally appropriate and relevant to the Bangladesh context.

1. **How has funding been used to support the local research team?**

Funding has been used to support the salaries of the icddr,b research team (MIH, SA, QR, MB, MT, BS, JH). MIH has been supported from icddr,b/WEHI to complete his PhD at the University of Melbourne.

1. **How are research staff who conducted data collection acknowledged?**

This paper is one of several from the EDIVA programme. Members of the icddr,b research team are either study authors or are acknowledged.

1. **Do all members of the research partnership have access to study data?**

Yes, all members of the research partnership have access to de-identified study data.

1. **How was data used to develop analytical skills within the partnership?**

As part of her PhD, data analysis for this study was completed by EV and guided by AM. EV and MIH completed two Stata training courses at the Murdoch Children’s Research Institute. MIH has analysed another component of the survey (submitted for publication) and developed his analytical skills through supervision from ED and SRP.

1. **How have research partners collaborated in interpreting study data?**

EV completed the data analysis to fulfil the requirements of her doctoral research. Discussions were then held with icddr,b colleagues to understand and develop the implications for research, including for the EDIVA programme, and for policy and practice.

1. **How were research partners supported to develop writing skills?**

The research team included senior academics (KHP, MAB, CV) who provided supervision for EV’s doctoral research. The icddr,b team (MIH, SA, QR, MB, MT, BS, JH) are researchers who have previously led or contributed to academic research articles. MIH is currently completing his PhD at the University of Melbourne. SA, BS, and JH will either be the lead or senior authors on publications arising from the EDIVA programme.

1. **How will research products be shared to address local needs?**

All papers resulting from the EDIVA programme will be published open access and results will be disseminated at relevant conferences. icddr,b research team members will meet with policymakers from the Bangladesh Ministry of Health and Family Welfare to share findings from the EDIVA programme.

1. **How is the leadership, contribution, and ownership of this work by LMIC researchers recognised within the authorship?**

EV led this work and is therefore the first and corresponding author. MIH (icddr,b/WEHI), ED (WEHI), and SA (icddr,b) had a key role in developing the survey and are second, third, and fourth authors, respectively. QR (icddr,b), MB (icddr,b), and MT (icddr,b) oversaw the data collection. JH (icddr,b) and SRP (WEHI) are the principal investigators of the EDIVA programme. BS (icddr,b) is the lead implementation scientist and assisted in interpreting the study findings to develop implications for research, policy, and practice.

1. **How have early career researchers across the partnership been included within the authorship team?**

The first author, EV, is a PhD candidate at the University of Melbourne and the study is part of her PhD research. MIH is also a PhD candidate at the University of Melbourne. BS, SA, ED, QR, MB, MT, and AM are early career researchers who have experience with academic writing and are interested in building their track record.

1. **How has gender balance been addressed within the authorship?**

Six authors are female (EV, ED, JH, CV, MAB, KHP) and eight authors are male (MIH, BS, SA, AM, QR, MB, MT, SRP)

1. **How has the project contributed to training of LMIC researchers?**

As mentioned in #3, MIH is completing his PhD as part of the EDIVA programme (supervised by ED, SRP).

1. **How has the project contributed to improvements in local infrastructure?**

This project has not directly contributed to improvements in local infrastructure.

1. **What safeguarding procedures were used to protect local study participants and researchers?**

Survey data were collected at the woman’s home, with women having the option to be accompanied by a family member, such as their mother-in-law. Women did not have to answer questions they did not want to answer and were able to stop the survey at any time. icddr,b research team members (field research assistants, field workers, country principal investigators) met regularly to debrief and address any issues arising during data collection.
