# Supplementary material for: Testing and treating anaemia in pregnant women in Bangladesh: a cross-sectional survey
Source: BMJ Public Health. 2025 Jul 16;3(2):e002167. doi: 10.1136/bmjph-2024-002167 (PMC12273131; doi:10.1136/bmjph-2024-002167)
Supplement: online supplemental file 4 [file bmjph-3-2-s004.docx]

**Online supplemental tables**

**Online supplemental table 1.** Percentage of pregnant women (n = 963) tested for anaemia in Narayanganj district, Bangladesh

| **Variable** | **Frequency (%)** |
| --- | --- |
| **Pregnant women tested for anaemia ^a^** | |
| Yes | 505 (52.4) |
| No | 458 (47.6) |

^a^ unsure (n = 37) excluded from denominator.

**Online supplemental table 2**. Percentage of pregnant women (n = 963) tested for anaemia in Narayanganj district, Bangladesh by social and equity dimensions

| **Percentage of pregnant women tested for anaemia** | **Observations** | **Unadjusted percentages and 95% CI** |
| --- | --- | --- |
| **Woman’s education level ^a^** | | |
| No education | 38 | 15.8 (95% CI: 6.0-31.3) |
| Primary education (1-8 years) | 509 | 45.4 (95% CI: 41.0-49.8) |
| Secondary education (9-12 years) | 376 | 63.0 (95% CI: 57.9-67.9) |
| Tertiary education (> 12 years) | 37 | 81.1 (95% CI: 64.8-92.0) |
| **Husband’s education level ^b^** | | |
| No education | 125 | 25.6 (95% CI:18.2-34.2) |
| Primary education (1-8 years) | 487 | 50.1 (95% CI: 45.6-54.6) |
| Secondary education (9-12 years) | 296 | 63.5 (95% CI: 57.7-69.0) |
| Tertiary education (> 12 years) | 52 | 76.9 (95% CI: 63.2-87.5) |
| **Household wealth index ^c^** |  |  |
| Poorest | 194 | 34.0 (95% CI: 27.4-41.2) |
| Second | 193 | 49.2 (95% CI: 42.0-56.5) |
| Middle | 200 | 52.5 (95% CI: 45.3-59.6) |
| Fourth | 187 | 55.6 (95% CI: 48.2-62.9) |
| Wealthiest | 186 | 72.0 (95% CI: 65.0-78.4) |
| **Person making decision about woman’s healthcare** | | |
| Woman and husband jointly | 572 | 52.3 (95% CI: 48.1-56.4) |
| Husband only | 214 | 50.0 (95% CI: 43.1-56.9) |
| Other (mother-in-law, mother, father-in-law, father, brother, brother-in-law, sister-in-law, not specified) | 105 | 50.5 (95% CI: 40.5-60.4) |
| Woman only | 72 | 63.9 (95% CI: 51.7-74.9) |

^a^ n = 960. ^b^ n = 960. ^c^ n = 960.

**Online supplemental table 3.** How pregnant women are being tested for anaemia within the Bangladesh health system (n = 505)

| **Variable** | **Frequency (%)** |
| --- | --- |
| **Health facility where anaemia test conducted ^a^** | |
| Private | 393 (77.8) |
| Tertiary | 51 (10.1) |
| Non-governmental organisation | 24 (4.8) |
| Secondary | 20 (4.0) |
| Primary | 16 (3.2) |
| Home | 1 (0.2) |
| **Healthcare provider who conducted the anaemia test** | |
| Medical technologist | 350 (69.3) |
| Nurse/Midwife/Paramedic | 113 (22.4) |
| Graduate doctor | 24 (4.8) |
| Unsure | 13 (2.6) |
| Community Health Care Provider | 3 (0.6) |
| Non-governmental organisation worker | 2 (0.4) |
| **Method of anaemia test** | |
| Venous full blood count | 484 (95.8) |
| Capillary colour scale | 10 (2.0) |
| Unsure | 6 (1.2) |
| Capillary HemoCue | 5 (1.0) |
| **Result of anaemia test** | |
| Not anaemic | 317 (62.8) |
| Anaemic | 148 (29.3) |
| Unsure | 40 (7.9) |

^a^ private health facilities (private practitioners chamber, private clinic/hospital, private medical college hospital). Primary health facilities (upazila health complex, union health and family welfare centre, satellite clinic, community clinic). Secondary health facilities (district/sadar hospital, mother and child welfare centre). Tertiary health facilities (public medical college hospital, tertiary hospital).
