# Supplementary material for: Testing and treating anaemia in pregnant women in Bangladesh: a cross-sectional survey
Source: BMJ Public Health. 2025 Jul 16;3(2):e002167. doi: 10.1136/bmjph-2024-002167 (PMC12273131; doi:10.1136/bmjph-2024-002167)
Supplement: Abstract translation 1 [file bmjph-3-2-s005.docx]

| **সারসংক্ষেপ (ABSTRACT)** |
| --- |
| **ভূমিকা:** গর্ভকালীন রক্তস্বল্পতা একটি গুরুত্বপূর্ণ জনস্বাস্থ্য সমস্যা, যা নিম্ন ও মধ্যম আয়ের দেশগুলিতে নারীদের উপর অসমভাবে প্রভাব ফেলে। বাংলাদেশে, ৩৮.৬% গর্ভবতী নারী রক্তস্বল্পতায় ভোগেন। যদিও গর্ভাবস্থায় রক্তস্বল্পতা প্রতিরোধ সম্পর্কে অনেক কিছু জানা গেছে, তবে নারীদের কীভাবে রক্তস্বল্পতা পরীক্ষার আওতায় আনা হয় এবং কীভাবে চিকিৎসা দেওয়া হয়, সে বিষয়ে তেমন তথ্য নেই। তাই এই গবেষণার লক্ষ্য ছিল বাংলাদেশে গর্ভবতী নারীদের রক্তস্বল্পতার জন্য কীভাবে পরীক্ষা করা হয়, এবং এতে সামাজিক ও সাম্যতাভিত্তিক পার্থক্য কীভাবে প্রতিফলিত হয়, তা বিশ্লেষণ করা। পাশাপাশি, রক্তস্বল্পতার চিকিৎসা ও গর্ভকালীন ইনট্রাভেনাস আয়রনের বা শিরায় আয়রন ইনজেকশনের (IV iron) ব্যবহার বিষয়েও অনুসন্ধান করা হয়।  **পদ্ধতি:** বাংলাদেশের নারায়ণগঞ্জ জেলায় পূর্বে কখনো গর্ভধারণ করেছিলেন এমন ১,০০০ জন নারীর অংশগ্রহণে সাক্ষাৎকারভিত্তিক একটি ক্রস-সেকশনাল জরিপ পরিচালিত হয়। মূল ফলাফলসমূহ ছিল রক্তস্বল্পতা পরীক্ষা, চিকিৎসা এবং ইনট্রাভেনাস আয়রনের ব্যবহার। আমরা বর্ণনামূলক বিশ্লেষণ করেছি এবং শিক্ষা, সম্পদ এবং নারীর স্বাস্থ্যসেবায় সিদ্ধান্তগ্রহণকারীর ভিত্তিতে রক্তাল্পতা পরীক্ষার শতকরা হার ও তার ৯৫% বিশ্বাসযোগ্য সীমা (কনফিডেন্স ইন্টারভাল) নির্ধারণ করেছি।  **ফলাফল:** গবেষণার আওতাধীন প্রায় অর্ধেক নারীর (৫০৫/৯৬৩, ৫২.৪%) পূর্ববর্তী গর্ভাবস্থায় রক্তস্বল্পতা পরীক্ষা করা হয়েছিল। পরীক্ষার হার ছিল কম যারা শিক্ষা পাননি (১৫.৮%, ৯৫% CI: ৬.০–৩১.৩), যাদের স্বামীর শিক্ষা নেই (২৫.৬%, ৯৫% CI: ১৮.২–৩৪.২), অথবা যারা দরিদ্রতম বা সর্বনিম্ন ধনসম্পদ স্তরের (lowest wealth quintile) অন্তর্ভুক্ত (৩৪.০%, ৯৫% CI: ২৭.৪–৪১.২)। রক্তাল্পতার জন্য নারীরা প্রায়শই বেসরকারি স্বাস্থ্যসেবায় পরীক্ষা করিয়েছেন (৩৯৩/৫০৫, ৭৭.৮%), যেখানে পরীক্ষাটি করেছেন একজন মেডিকেল টেকনোলজিস্ট (৩৫০/৫০৫, ৬৯.৩%) এবং ভেনাস ফুল ব্লাড কাউন্ট (৪৮৪/৫০৫, ৯৫.৮%) পদ্ধতি ব্যবহার করা হয়েছে। যেসব নারীদের রক্তাল্পতা ধরা পড়েছে, তাদের প্রায় সবাই চিকিৎসা পেয়েছেন (১৩৫/১৪২, ৯৫.১%)। পূর্ববর্তী যেকোনো গর্ভাবস্থায়, মাত্র ৩.৪% নারী ইনট্রাভেনাস আয়রন (শিরায় আয়রন ইনজেকশন) গ্রহণ করেছেন (৩৩/৯৮৫)।  **উপসংহার:** গবেষণায় দেখা গেছে যে গর্ভাবস্থায় বাংলাদেশের সকল নারীর রক্তস্বল্পতার পরীক্ষা নিশ্চিত করার জন্য যথেষ্ট বিনিয়োগের প্রয়োজনীয়তা রয়েছে। এটি গর্ভকালীন সেবা গ্রহনের সময় (অ্যান্টিনেটাল কেয়ার) স্বাস্থ্যকেন্দ্রে পরীক্ষা বাড়ানোর মাধ্যমে অর্জন করা যেতে পারে। তবে, স্বাস্থ্যকেন্দ্র এবং স্বাস্থ্যকর্মীদের এই পরীক্ষার ফলে বাড়তি রোগী শনাক্ত হওয়ার জন্য প্রস্তুত থাকতে হবে। |
